# Health system constraints affecting treatment and care among women with cervical cancer in Harare, Zimbabwe

**DOI:** 10.1186/s12913-019-4697-6

**Published:** 2019-11-12

**Authors:** O. Tapera, G. Dreyer, W. Kadzatsa, A. M. Nyakabau, B. Stray-Pedersen, Hendricks SJH

**Affiliations:** 1 School of Health Systems and Public Health, University of Pretoria, Pretoria, South Africa; 2 Gynaecologic Oncology, Department of Obstetrics and Gynaecology, University of Pretoria, Pretoria, South Africa; 3 Parirenyatwa Group of Hospitals, Radiotherapy Centre, Harare, Zimbabwe; 4 Institute of Clinical Medicine, University in Oslo and Womens’ Clinic, Oslo University Hospital, Oslo, Norway; 5 Faculty of Dentistry and Oral Health Hospital, Sefako Makgatho Health Sciences University, Pretoria, South Africa; 6 Faculty of Health Sciences, University of Fort Hare, East London, South Africa

**Keywords:** Cervical cancer, Health system, Constraints, Access, Treatment and care, Sequential explanatory mixed methods, Policies, Harare

## Abstract

**Background:**

Cervical cancer is a major cause of morbidity and mortality among women yet access to treatment and care remains a huge challenge in Zimbabwe. The objective of this study was to investigate health system constraints affecting engagement into treatment and care by women with cervical cancer in Harare, Zimbabwe.

**Methods:**

A sequential explanatory mixed methods design was used for this study. Phase 1 comprised of two surveys namely: patient and health worker surveys with sample sizes of 134 and 78 participants respectively. Validated structured questionnaires programmed in Android tablet with *SurveytoGo* software were used for data collection during the surveys. Univariate analyses were conducted using *STATA®* version 14 to generate descriptive statistics. In phase 2, 16 in-depth interviews, 20 key informant interviews and 6 focus groups were conducted to explain survey results. Participants were purposively selected and sample sizes were informed by saturation principle. Participants in phase 1 and 2 were different. English transcripts were manually coded line by line in *Dedoose software* using the thematic codes that had been established from the survey data. The final codes were used to support and explain the survey data at the interpretation stages.

**Results:**

Health system constraints identified in surveys were: limited or lack of training for health workers, weakness of surveillance system for cervical cancer, limited access to treatment and care, inadequate health workers, reliance of patients on out-of-pocket funding for treatment services, lack of back-up for major equipment. Qualitative inquiry revealed the following barriers to treatment and care: high costs of treatment and care, lack of knowledge about cervical cancer and bad attitudes of health workers, few screening and treating centres located mostly in urban areas, lack of clear referral system resulting in bureaucratic processes, and limited screening and treating capacities in health facilities due to lack of resources.

**Conclusion:**

The results of this study show that health system and its organization present barriers to access of cervical cancer treatment and care among women. Strong political will, mobilization of resources both domestically and from partners in addition to sound policies are imperative to address key health system challenges.

## Background

Cervical cancer is the fourth most commonly diagnosed neoplasm among women worldwide [1]. The incidence of cervical cancer has been reported to vary by region, with Eastern and Southern parts of Africa carrying the largest burdens [2]. In Zimbabwe, cervical cancer is the most commonly diagnosed cancer among women and its high incidence is in tandem with the high HIV prevalence in the population [3]. At least 2270 cases of cervical cancer cases are reported yearly and about 1451 deaths attributable to cervical cancer are recorded annually [1]. Despite the fact that cervical cancer is preventable through early detection and treatment, most sub-Saharan African countries’ health systems are weak such that most cases present in late stages when incurable and only suitable for palliative care. Diagnosis and treatment of cervical cancer requires a health system which is functional, interactive and responsive physically and in terms of human resources to be able to identify, diagnose and treat cases. The infrastructure and organization of health systems in low-to-middle income countries are underdeveloped to provide comprehensive care to cancer patients [3]. Cervical cancer is a complex condition which requires continuum of care and comprehensive programmes which are also dependent on the stage of the cancer to improve health outcomes [4]. In low-to-middle income countries the morbidity and mortality rates of cervical cancer are high owing to late presentation of cases and failure to complete treatments due to lack of resources. Challenges of affordability, availability of drugs and accessibility of treating facilities impede engagement of patients into treatment and care [3, 5].

Several years of economic challenges have reduced the capacity of the health system to provide comprehensive care for cervical cancer in Zimbabwe [4–6]. Health policies in the country are developed to address access and distribution of healthcare services but they do not address key components as reported by Navarro [7]. The most recent or current National Cervical Cancer policies did not outline key determinants of access; nor provide mechanisms of addressing known barriers to access to treatment and care. Understanding that generally cancer occurs in a complex social, political, cultural, environmental and economic context is critical in investigating the possible barriers and promoters of access and utilization of treatment and palliation services [8]. Current evidence from a recent study suggests that barriers to access and use of cancer care persist up until death [9]. Reports of palliative care use have concluded that older patients are less likely to receive these services compared to their younger counterparts [8, 10]. In Zimbabwe, healthcare access and utilization entrenched by age, social class, income, occupation, ethnicity, gender and place of residence are well documented [11]. However, there is no documented evidence of how the functioning of the health system and its organization are contributing barriers to cervical cancer treatment and care. This study was conducted to address that knowledge gap in the country.

## Methods

This study was a sequential explanatory mixed methods design and it was conducted in Harare, Zimbabwe. The study was in two distinct phases; phase 1 which was quantitative and comprised of two descriptive cross sectional surveys and phase 2, which comprised of qualitative inquiry. Qualitative phase was used to explain results from the descriptive cross sectional surveys and findings were integrated at interpretation stage.

### Data sources and population

#### Phase 1

Two cross sectional surveys were conducting namely: 1) Patient survey 2) Health worker survey. The surveys were conducted in health facilities providing treatment and care for cervical cancer patients in Harare namely; Harare Hospital, Parirenyatwa Hospital and Island Hospice. Both patients and health workers were selected randomly using health facility records as sampling frames. Patients who visited the study sites during January–April 2018 with the following characteristics: histologically confirmed squamous cell carcinoma of the cervix as shown by their records or verified by health workers, at least 25 years of age, stable to be interviewed as approved by their clinicians and consented in writing were enrolled into the survey. The age range was informed by studies and anecdotal evidence from health facilities that have shown that the incidence of cancer is higher among women who are older than 30 years. The Zimbabwe Cervical Cancer Policy recommends routine screening for sexually active females between 18 and 65 years old [12, 13] and for that reason it was plausible to recruit participants aged at least 25 years old for this study. During the study no women with cervical cancer aged less than 30 years were identified across the three study sites. A total of 134 patients with different cervical cancer disease stages were enrolled in the study. Patients were selected regardless of where they were resident. This is because the chosen health facilities were tertiary facilities that treat and care for patients from across the country and for this study it was important to reduce bias by selecting patients regardless of where they stayed. For the health worker survey, 78 participants were selected randomly from the health facilities from which cervical cancer participants were drawn. Staff registers were used as the sampling frame from which participants were selected to avoid bias by selecting from those on duty at the time of the survey. Validated structured questionnaires were used for data collection for both surveys (see Additional files 1 & 2) [14]. Questionnaires were programmed in *SurveytoGo* software in an Android tablet to allow for electronic data collection.

#### Phase 2

In the qualitative inquiry, participants were purposively selected for enrolment in the study. Participants were selected in communities, health facilities and in institutions involved with cervical cancer interventions. Focus group discussions, in-depth and key informant interviews were conducted using discussion and interview guides developed based on the survey findings (see Additional file 3). For FGDs and in-depth interviews the participants were healthy men and women, caregivers and patients selected from communities and health facilities. Key informants were selected from health facilities, Ministry of Health and Child Care Family Health directorate, non-governmental organizations (NGOs) involved with cervical cancer programmes, World Health Organization, churches, and traditional healer’s associations. A total of 84 participants were selected based on desired characteristics for the study. Six focus groups with an average of 8 participants each were conducted in the communities and one of the treating health facilities (Parirenyatwa Hospital). For in-depth and key informant interviews 16 and 20 participants were enrolled for participation based on theoretical saturation principle [15]. All interviews and FGDs were audio-recorded after obtaining written consent from the participants.

### Data analysis

#### Phase 1

Data from the two surveys were analyzed separately as the variables were not related. This study focused and reported on the key health service attributes. Variables related to the health system for cervical cancer were selected from the two datasets and analyzed. Univariate analysis was conducted for both surveys using datasets that had been cleaned prior to analysis. Data cleaning process involved combining some responses with low frequency such as “*Frequency of being seen by specialists in previous 6 months*” and removing data from pilot interviews which was not included in this analysis. All analyses were conducted using *STATA* version 14 (StataCorp, Texas).

#### Phase 2

Transcription and translation of audio-recordings were undertaken by the researcher and his assistants. Uniquely identified transcripts were analyzed using themes that had been established apriori based on the survey findings. Unique identifiers were used to link the guides and the interview or FGD only after the conclusion of transcription. All in-depth interviews, key informant interviews and FGDs were coded manually line by line by the researcher using *Dedoose software* using the established thematic codes. Only five themes were identified as relevant to answer the research question for this study. Findings of phase 1 (surveys) and phase 2 (qualitative inquiry) were integrated at interpretation stage in which case qualitative findings assisted in explaining and interpreting survey results.

## Results

### Phase 1: quantitative findings

Table 1 shows that the mean ages of women with untreated and treated cervical cancer were 50.2 (SD = 10.8) and 52.9 (SD = 12.7) years respectively. Among women with untreated cervical cancer, 64% lived in urban areas while 36% resided in rural areas. For women with treated cervical cancer, 51 and 49% lived in urban and rural areas respectively. There were few but notable differences between treated and untreated women with cervical cancer with respect to the selected health service attributes. The proportion of women with treated cervical cancer who were seen once or more times by specialists in the previous 6 months was higher than for untreated women. These interfaces with specialists included treatment and review visits. Women with untreated cervical cancer had less access to regular general practitioners compared to women with treated cervical cancer. Women with untreated cervical cancer were less likely to know the estimated distances from their residence to the nearest cervical cancer screening health facilities compared to treated women.

**Table 1:** Demographic characteristics, health service attributes and their distribution amongst women with cervical cancer

Participant type	Cervical cancer patients [*N* = 134]	
Variables	Untreated [*n* = 42] (%)	Treated [*n* = 92] (%)
Age		
Mean (years)	50.2	52.9
Area of residence		
Urban	27 (64)	47 (51)
Rural	15 (36)	45 (49)
Method of payment for health services		
Medical aid (insurance)	5 (12)	22 (24)
Out-of-pocket	37 (88)	70 (76)
Distance from nearest health facility (km)		
≤ 10	38 (90)	76 (82)
11–20	4 (10)	8 (9)
> 20	0	8 (9)
Mode of transport to nearest health facility		
Walking	28 (67)	49 (53)
Public transport	13 (31)	33 (36)
Private car	1 (2)	9 (10)
Motorcycle	0	1 (1)
Time to travel to nearest health facility (minutes)		
≤ 30	32 (76)	75 (82)
31–60	7 (17)	12 (13)
> 60	3 (7)	5 (5)
Distance from nearest cervical cancer screening health facility (km)		
≤ 10	5 (12)	30 (33)
11–50	4 (10)	18 (19)
> 50	1 (2)	7 (8)
Don’t know	32 (76)	37 (40)
Mode of transport to nearest cervical cancer screening health facility		
Walking	3 (7)	12 (13)
Public transport	35 (83)	70 (76)
Private car	3 (7)	10 (11)
Other	1 (3)	0
Time to travel to nearest screening health facility (minutes)		
≤ 30	15 (36)	51 (45)
31–60	14 (33)	25 (27)
> 60	9 (22)	10 (11)
Don’t know	4 (9)	6 (7)
Access to Specialists		
Yes	34 (81)	73 (79)
No	8 (19)	19 (21)
Frequency of being seen by Specialists in previous 6 months		
None	17 (40)	27 (29)
Once or more	25 (60)	65 (71)
Access to a regular General Practitioner		
Yes	8 (19)	56 (61)
No	34 (81)	36 (39)
Visits to health facility or doctor in previous 6 months		
None	10 (24)	4 (4)
≤ 10 times	30 (71)	55 (60)
11–20 times	2 (5)	17 (19)
> 20	0	16 (17)
Challenges experienced in seeking treatment.		
Finances	20 (48)	45 (49)
Transport	22 (52)	39 (42)
Other	0	8 (9)

Table 2 shows that the mean age of health workers who were enrolled in this study was 37.3 years (SD = 10), while the mean number of years of experience was 11.5 years (SD = 10). The subsamples for Harare hospital, Parirenyatwa Hospital and Island Hospice were 26, 42 and 10 respectively. The table also shows that health worker perceptions or knowledge of most of the health service attributes related to cervical cancer treatment and care investigated in the study. Twenty-eight percent (28%) of health workers reported not having received training and adequately trained for cervical cancer treatment and care. Thirty-eight percent (38%) reported not knowing or having read both the National Cancer Prevention and Control Strategy (2013–2017) and the Cervical Cancer Prevention and Control Strategy for Zimbabwe (2016–2020). The proportion of health workers who perceived that health professionals were not enough to meet the demand of services in health facilities was 88% compared to 12% who believed they were sufficient. Only 47% of health workers perceived that most women with cervical cancer had access to treatment and care. The proportion of participants who believed that the surveillance system for cervical cancer was weak was 77%. However, despite these findings 69% of health workers were motivated to provide their services and only 26% reported poor working conditions in the health facilities. The majority (63%) of participants reported that the main mode of payment for cervical cancer treatment and care was out-of-pocket financing and the same proportion reported that the major barrier to treatment was lack of finances. In addition, 59% of the health workers suggested free treatment for cervical cancer as a solution to improving access in the country. While 58% of participants reported having adequate basic equipment in health facilities, as high as 78% reported unavailability of back-up for major equipment.

**Table 2:** Characteristics and health service attributes of health workers and facilities providing treatment and care for cervical cancer

Variable	Number of health workers [*N* = 78] (%)
Age	
Mean (years)	37.3
Gender	
Male	15 (19)
Female	63 (81)
Years of experience	
Mean (years)	11.5
Health Facilities	
Harare hospital	26 (33)
Parirenyatwa hospital	42 (54)
Island Hospice	10 (13)
Received on-the-job training for cervical cancer treatment or palliative care	
No	22 (28)
Yes	56 (72)
Perception of being adequately trained to provide cervical cancer treatment and palliative care	
No	22 (28)
Yes	56 (72)
Health facilities have clinical guidelines for cervical cancer treatment and palliative care	
No	59 (76)
Yes	19 (24)
Have knowledge or read National Cancer Prevention and Control Policy (201342018)	
No	30 (38)
Yes	48 (62)
Have knowledge or read Zimbabwe Cervical Cancer Prevention and Control Strategy (2016–2020)	
No	30 (38)
Yes	48 (62)
Perceptions of adequacy of policies and strategies for treatment and care of cervical cancer patients	
No	44 (56)
Yes	34 (44)
Perceptions of strength of surveillance system for cervical cancer	
No	60 (77)
Yes	18 (23)
Motivated to provide services	
No	24 (31)
Yes	54 (69)
Working conditions	
Excellent	4 (5)
Good	54 (69)
Poor	20 (26)
Perceptions of adequate number of health workers	
No	69 (88)
Yes	9 (12)
Perceptions of histological investigation capacity from suspected cervical cancer patients	
No	33 (42)
Yes	45 (58)
Perceptions of access to treatment and care by patients	
No	41 (53)
Yes	37 (47)
Payment methods for services by majority of patients	
Out-of-of pocket	49 (63)
Medical aid	26 (33)
Social Welfare	3 (4)
Challenges faced by most patients in seeking treatment and care	
Transport	29 (37)
Finances	49 (63)
Proposed solutions to improve access to treatment and care for cervical cancer	
Free treatment	46 (59)
Built more treating facilities	13 (17)
Government to assist people to seek treatment abroad	8 (10)
Increase human resources capacity	7 (9)
Other	4 (5)
Perceive adequate basic equipment available	
No	33 (42)
Yes	45 (58)
Perceive functional equipment available	
No	42 (54)
Yes	36 (46)
Perceive back up of major equipment available	
No	61 (78)
Yes	17 (22)
Perceive analgesic stock-outs of at least once in previous 3 months	
No	61 (78)
Yes	17 (22)
Perceive contingency plans for major drugs for cervical cancer available	
No	19 (24)
Yes	59 (76)

### Phase 2: qualitative findings

#### Main theme: health system is constrained to provide cervical cancer treatment and care

The main theme that emerged from this study was that the functioning and organization of the health system was compromised to provide comprehensive treatment and care to women with cervical cancer. The salient sub-themes that emerged are reported as quotes below:

#### Sub-theme: high cost of treatment and care

Most participants reported that the costs of treatment and care for cervical cancer were exorbitant to be afforded by ordinary patients. The costs included staging and monitoring investigations which are provided in private facilities as public health facilities do not have sufficient capacity to offer them. Treating health facilities do not provide chemotherapy drugs and they refer patients to private pharmacies. Since these drugs are imported the costs of buying them is a huge burden on the patients. Furthermore, lack of price regulatory framework for healthcare commodities in the country subjects patients to the mercy of private health workers. These issues are revealed in the following statements from some participants:

*“Not everyone can manage because the costs of treatment here are high”.* Caregiver from Chishawasha

*“From the little knowledge that l have it looks like they are not accessing the treatment because of the economic situation. Medication is expensive and not everyone is on medical aid and for those who are it does not cover most illnesses and one will have to pay for the shortfalls”.* Key informant, Pastor

*“ … ..cancer medication tends to be more expensive … .”*Key informant, Pharmacologist

*“In most of our communities people don’t earn that much they can’t afford and therefore they end up seeking alternative therapies in the form of traditional healers or spiritual healers”.* Key informant, Gynaecologist

One cervical cancer patient in an FGD reported that:

*“I was prescribed a drug at Parirenyatwa and was told to go and buy in pharmacies but I couldn’t afford. I didn’t buy any of the prescribed drugs as I did not have the money”*

#### Sub-theme: limited health professional’s knowledge and attitudes

Some respondents especially key informants reported that health workers still lacked adequate knowledge about cervical cancer to educate the communities and also to know what to do when a patient presents with symptoms. Some of the symptoms may not be so obvious and health workers especially in the primary care health facilities may not suspect cervical cancer or refer someone for screening. Some participants reported bad attitudes amongst health workers for which the unintended consequences are poor health seeking behaviours among the communities.

*“One may just think of the queues that will be there and also it depends with the nurses that are on duty because some of them aren’t friendly, they are harsh so one may decide to just go back home without being screened for cervical cancer.”* Healthy woman from Hopely

*“Some people go to private doctors so as to get quick assistance and in some cases one gets treatment for a different disease, so instead of screening for cancer first one maybe diagnosed with fibroids and they will start treating fibroids yet the patient has cervical cancer.”* Caregiver from Goromonzi.

“*… .. if one goes to the doctors and is given wrong diagnosis and treatment the cancer would be spreading therefore contributing to advanced disease presentation. In my case I was discharging some odourless fluids and I went to the doctors and they started treating me for STIs yet the test results were negative for STIs”* Cervical cancer survivor from Mabvuku

*“You walk into a rural health facility and you ask nurses about cervical cancer or cancer in general but they have no clue of what it is but we are asking people in the communities to go to the clinic where the nurses don’t know anything about cancer.”* Key informant, Midwife.

#### Sub-theme: unclear pathway of care

Most participants revealed that the referral systems being used for cervical cancer patients were bureaucratic and could result in attrition of patients or incentivizing them to seek alternatives such as traditional medicines and spiritual interventions. Some participants reported that by the time the patients get to be engaged into treatment and care they would have interfaced with many health facilities or service providers and their disease would have advanced.

A caregiver from Goromonzi said:

*“The challenges we faced include the following: we went to different hospitals, consulted different traditional healers, prophets and went to different churches to no avail my mother would get prayed for and feel better for maybe 3 days and it would worsen. That is why we got to a point of believing that there were evil spirits behind my mothers’ sickness. At Karanda hospital they did a biopsy and after 2 weeks her results were out and then she was referred to Parirenyatwa hospital. That is when we came in March and the doctors were on strike but it seemed my mother was getting worse by the day after the biopsy was taken”.*

One cervical cancer patient from Kwekwe also reiterated the challenges of the referral system:

*“The fact that when you come here you will have to pay consultation fees, money is needed for every service they will render to you, like scan, blood tests, blood transfusion etc. However; the costs are lower with prophets or traditional healers”.*

A cervical cancer patient from Harare reported in a FGD that:

*“I was screened at Parirenyatwa in 2013 and I was operated and they took specimen after the operation and told me that I was now okay. From 2013 I considered myself as a cancer free person then last year I started to feel pain and I visited the doctor. I was told that the pain was being caused by ulcers. Around March last year (2017) I started spotting and I visited my nearest clinic and consulted them about my issue and I was referred to a gynecologist who took some specimens and I was told that the cervical cancer had recurred”*

#### Sub-theme: limited capacities of screening and treating health facilities

Some participants reported challenges in accessing cervical cancer screening services resulting in discouragement from seeking such services in future. Most participants reported that even though treatment was being offered in Harare, some patients could not access as they lived in far away places. Some reported that treating health facilities did not have capacity to conduct all the staging and monitoring investigations and provide the drugs that were needed by patients mostly chemotherapies and analgesics for pain management. Most participants particularly patients and health workers reported that the radiotherapy machine had frequent breakdowns and there is no back-up for this machine as it is a very expensive piece of equipment.

One oncologist reported that:

*“I think also the fact that access to treatment was not consistent in the sense that there were times when machines for treating patients were not working that also can be a negative push factor.”*

A cervical cancer patient from Beatrice also added to the challenge of limited capacities in health facilities by reporting that:

*“When l first went (for screening) the nurses were on strike, I then went back there twice more and they said they had no equipment, that is when l was referred to private health facility where I had my biopsy done and it was taken to private laboratory.”*

Another patient from Chiredzi reported some of the challenges experienced in receiving treatment:

*“Every Monday I have my blood examined and then Tuesdays I would go for chemotherapy but sometimes I didn’t have the money to buy the medication for that particular session so I would have to look for money and then go later.”*

## Discussion

This study has revealed a myriad of health system barriers to accessing treatment and care by women with cervical cancer in Zimbabwe. Some of the structural barriers found in our quantitative study were: limited or lack of training for health workers, weakness of surveillance system for cervical cancer, limited access to treatment and care, inadequate health workers, reliance of patients on out-of-pocket funding for treatment services, lack of back-up for major equipment. The barriers to cervical cancer treatment and care reported in the qualitative study included: high costs of treatment and care, lack of knowledge about cervical cancer and bad attitudes of health workers, few screening and treating centres located mostly in urban areas, lack of clear referral system resulting in bureaucratic processes, and limited screening and treating capacities in health facilities due to lack of resources. These barriers were not only in the lack of infrastructure and resources to provide comprehensive services but in the organization of the health system. These findings are supported by a recent review of sub-Saharan Africa which revealed major challenges in cancer care. Researchers reported that diagnosis and treatment of cancer require a health system that is functional, interactive and responsive physically and has adequate human resources to identify, diagnose and treat cases. The elements of such a health system are: physical infrastructure, qualified staff, supportive policies, and supportive laws [3].

Our findings suggest that the frequency of interface with specialists and access to regular general practitioners may be independent predictors of engagement into treatment and care. Limited access to health professionals may explain the relatively low uptake of treatment and care services among women with cervical cancer resulting in presentation of advanced disease [4, 5]. In Zimbabwe only about 10% of the population is on medical aid [16] and therefore access to regular general practitioners and specialists may be limited as most people rely on local primary care clinics for any health issue. These findings are consistent with those of a Pacific region study which reported limited number of cancer specialists due to lack of resources and migration to “greener pastures”. Access to cancer treatment was reported as limited in the Pacific region as treatment centres were far away and patients were unwilling to travel long distances to seek screening and treatment services [17]. Our results are also supported by the reports of recent studies conducted in Zimbabwe which showed health system challenges to provide comprehensive treatment and care for cervical cancer [4, 5].

Our study revealed that some women with cervical cancer were never screened as they presented with clinically advanced disease and were straightway referred for histological investigations. Some of these women were living with HIV/AIDS who are recommended for screening yearly due to the high risk of cervical cancer incidence in this population. Zimbabwe adopted the integration of sexual reproductive health (SRH) services including cervical cancer screening with HIV/AIDS services to improve access to screening by PLWHA [13]. Surprisingly our findings revealed that some of these patients presented with locally advanced disease despite the integration of services in the country. This suggests noteworthy health system or organization or policy failures. Furthermore, screening centres are still limited and are available mostly in urban centres yet the majority of women in Zimbabwe live in rural areas [18]. Due to lack of financial resources most women cannot travel to seek health services for screening and later on for treatment. These findings are in tandem with those of Mohammadnezhad et al. [17] and Kuguyo et al. [5] who reported limited and centralized screening and treatment services in low-income contexts.

Our study revealed suboptimal training and knowledge for the treatment and care of cervical cancer patients among health workers in tertiary health facilities. This was supported by the fact that only 62% of the health workers from tertiary health facilities knew or had read the key national cancer policies. Lack of knowledge among health workers particularly in the primary health care system is manifested in wrong diagnoses and treatments of patients leading to late disease presentations [4, 5]. Bad attitude towards patients with chronic diseases by health workers play a significant role in undermining health seeking behaviors [19] and may lead to patients resorting to alternative interventions. Unavailability of adequately trained health workforce reduces the availability of diagnosis and treatment services [17, 20]. Studies in the USA have reported that deficiency of knowledge and lack of training of health workers were barriers to cancer services supporting our findings [20, 21]. Lack of knowledge and inadequate training in cancer among health workers in tertiary facilities is real in Zimbabwe and this suggests even bigger gaps in the primary healthcare system.

Findings from this study showed that cervical cancer surveillance system in the country is weak and cannot account for some cases of cervical cancer and it is probable that the majority of cases are not reported or documented. Considering the limited capacities in screening facilities and the bureaucracy involved in patient referrals to treatment centres suggested by our qualitative study, the burden of cervical cancer in Zimbabwe may be grossly underestimated. In addition, pathways to care are not clear and it takes more than just the patient and their immediate family to navigate through the multitudes of barriers to get into treatment. This could also explain late disease presentations and poor cervical cancer survival or outcomes [4, 5, 22]. The costs associated with transport to health facilities, investigations, purchasing of drugs mostly from private pharmacies and receipt of radiotherapy are so high that the majority of patients cannot afford them. The major barriers reported in our work were lack of transport and financial resources to pay for transport to health facilities, investigations, drugs and radiotherapy session. The other key limitation in the provision of cervical cancer treatment was technical breakdowns of the radiotherapy machines and lack of back-up equipment. This results in disrupted services and may explain loss to follow-up that was reported in our study. Most patients on radiotherapy require 21–23 sessions which translates in days to about a month of visiting the health facility for treatment and because the majority are not resident in Harare or do not have close relatives in Harare, any breakdown of the machines may discourage them from continuing with treatment leading to defaulting or loss to follow-up. Concomitant with lack of a follow-up system, this will result in high recurrence rates and poor survival from cervical cancer [5, 22].

Despite the multi-dimensional health system barriers to cervical cancer treatment and care, health workers were still hopeful and motivated to provide services to patients. However, several health worker strikes have occurred to pressure the government for better remuneration and working conditions, even during the course of this study. These strikes were also reported to have affected the treatment and care of cervical cancer patients. With the deterioration of working conditions, experienced health professionals may seek for better opportunities in foreign countries [6, 17]. This may result in understaffing and poor quality services in the country’s tertiary facilities compounding to the plight of women with cervical cancer. Nyazema [6] and Makoni [23] reported the devastating effects of economic hardships on the quality of health care services. Therefore, health systems challenges are indeed a barrier to accessing cervical cancer treatment and care among women and may be persistent in the recent to medium term future given the challenging macroeconomic milieu.

This study encountered some limitations worthy of mentioning; firstly we relied on descriptive analyses for our surveys and care must be exercised due to the risk of confounding or interaction effects. Secondly, this study was conducted in Harare and the results may not be generalizable to other settings. Most of the cervical cancer services (preventive, diagnostic, treatment and palliative care) are fairly available in Harare which is the capital of Zimbabwe and this is not the case across the country. Thirdly, this research involved descriptive cross sectional surveys whose findings cannot be used to infer causal relationships. However, this study had its fair share of strengths; the use of mixed methods design allowed for deeper understanding of issues and the involvement of diverse study populations resulted in a diversity of perspectives which is crucial in any research endeavor. Most importantly, to our knowledge, this study was the first primary research to investigate health system constraints affecting cervical cancer treatment and care among women in Zimbabwe.

## Conclusion

The results of this study show that the prevailing health system and its organization present barriers to the uptake of cervical cancer treatment and care. It is imperative that sustainable and workable strategies are devised and implemented, in the short-term future, in order to reduce the morbidity and mortality of cervical cancer in a low-income setting. Improvement in the organization and distribution of cervical cancer screening and treatment services need to address the identified barriers. Strong political will and mobilization of resources both domestically and from global partners to build more capacity for screening and treatment of cervical cancer will go a long way in mitigating some of the key health system challenges. Sound and realistic health policies that will create a health system that is functional, interactive and responsive to identify, diagnose and treat cases are recommended in addressing the growing incidence of cervical cancer among women in low-income countries. Capacity building of health care workers to improve their knowledge on cervical cancer is an imperative strategy low-income countries should consider.

## Supplementary information

**Additional file 1.** Validated structured questionnaire for healthy women and cervical cancer patients [English & Shona].

**Additional file 2.** Validated structured questionnaire for health workers [English].

**Additional file 3.** In-depth interview guide [English and Shona].

## Data Availability

The datasets used and/or analyzed during the current study are available from the corresponding author on reasonable request.

## Abbreviations

AIDS: Acquired immunodeficiency syndrome
FGD: Focus Group Discussion
HIV: Human immunodeficiency virus
NGO: Non-governmental organization
PLWHA: People living with HIV and AIDS
SRH: Sexual reproductive health
VIAC: Visual inspection with acetic acid cervicography
ZDHS: Zimbabwe demographic and health survey
