# Supplementary material for: Health system constraints affecting treatment and care among women with cervical cancer in Harare, Zimbabwe
Source: BMC Health Serv Res. 2019 Nov 12;19:829. doi: 10.1186/s12913-019-4697-6 (PMC6852958; doi:10.1186/s12913-019-4697-6)
Supplement: Supplementary file 1 — Additional file 1. Validated structured questionnaire for healthy women and cervical cancer patients [English & Shona]. [file 12913_2019_4697_MOESM1_ESM.docx]

**­­­­­­­­­­­­­­****COMMUNITY AND PATIENT SURVEY**

**Study title: Equity in access and utilization of cervical cancer treatment and palliation services in Harare, Zimbabwe.**

**SPEAK TO THE HEAD OF THE HOUSEHOLD:** Hello. My name is Oscar Tapera and I am a PhD student at the University of Pretoria, South Africa. We are interviewing women aged at least 25 years old here in [name of PLACE] in order to obtain your views,opinions and experience on Cervical Cancer treatment and palliative care services in Harare. You or your household has been randomly selected to participate in this study. This interview will take not more than 45 minutes and you are free to ask me any questions after the interview or if you need any clarity you may stop me during the interview so that I may assist you.

*Makadii. Zita rangu ndinonzi Oscar Tapera uye ndirikuita zvidzidzo zve PhD ku yunivhesiti yePretoria ku South Africa. Tiri kuita hurukuro nevanhukadzi vane makore makumi maviri nemashanu vanogara muzvimbo ino --------------- kuitira kuti tiwane maonero mapfungiro uye zvamakasangana nazvo pamusoro pe kurapwa kwe gomarara remuromo wechibereko pamwe nekuchengetwa kunoitwa vanhu vanegomarara iri mu Harare. Imi ne mhuri yenyu masarudzwa pachishandiswa nzira yemabhiza kuti mupinde mutsvakurudzo ino. Atitarisire kuti hurukuro yatichaita nemi itore maminetsi anopfura makumi mana nemashanu. Shure kwehurukuro iyi makasununguka kubvunza chero mibunzo yamuinayo kana kuti kune zvamunenge musina kunzwisisa makasununguka kundimisa mobvunza henyu kuitira kuti ndikubatsirei*.

**For household interviews speak to household head:** I would like your permission to identify a respondent and begin the interview. *Ndinokumbira mvumo yenyu kuti ndisarudze munhu mumwe chete wandingaite hurukuro naye.*

[**Note**: Ensure formal consent process is done prior to interviewing the selected respondent].

| ID01  ID02  ID03  ID04  ID05  ID06  ID07  ID08 | IDENTIFICATION Questionnaire No. [ ]  Province of residence : ______________________________________  District of residence : ______________________________________  Ward of residence : ________________________________________  Area of residence : __________________________________________  Health facility Name: __________________________________________  For health facility **Private -----------------------------1**  **Public-------------------------------2**  Type of respondent **Healthy woman -----------------1**  **Cervical cancer survivor----2** |
| --- | --- |
| ID09 | INTERVIEWER VISITS A B C  Visit 1 Visit 2 Visit 3  DATE ____________ ____________ ______________ |
| ID10  ID11  ID12 | HOUSEHOLD SELECTION STATUS Originally selected household ---------------------------------------1 (SKIP TO ID11)  Replacement -------------------------------------------------------------2  (If a replacement) REASON FOR REPLACEMENT  Not at home after repeated visits------------------------------------1  No eligible respondent--------------------------------------------------2  Refused --------------------------------------------------------------------3  Other (Specify)------------------------------------------------------------4  INTERVIEW STATUS  Completed interview-----------------------------------------------------1  Partially completed interview------------------------------------------2 |

| **SECTION 1. POPULATION CHARACTERISTICS** | | | | Skip instructions |
| --- | --- | --- | --- | --- |
| Q101 | How old were you at your last birthday?  *Ma/Waive nemakore mangani pabhavhadhe renyu/rako rekupedzisira?* | _____________ | Age in completed years |  |
|  |  |  |  |  |
| Q102a | What is your ethnicity?  *Urimurudzii* | Shona  Ndebele  Other African  White  Asian  Other (specify)___________ | 1  2  3  4  5 |  |
| Q102b | What is your current marital status?  *Makamira sei panhau dzewanano?* | Married/Co-habiting  Never married  Widowed  Divorced  Separated | 1  2  3  4  5 |  |
| Q103a | What is the highest level of school you attended: primary, secondary or higher?  *Ndechipi chinhanho chepamusoro mukudzidza chamaka pedza?* | Primary  Secondary  Higher  None | 1  2  3  4 |  |
| Q103b | What is the highest level of school attended by head of your household?  *Ndechipi chinhanho chapamusoro mukudzidza chakasvikwa ne anotungamira musha?* | Primary  Secondary  Higher  None  Not applicable | 1  2  3  4  5 |  |
| Q104a | Do you have any child(ren)?  *Mune mwana/vana here?* | Yes  No | 1  2 | If No skip to Q105 |
| Q104b | If **Yes**, how many children do you have?  *Kana ati hongu, Mune vana vangani?* | [ ] | Number of children |  |
| Q105 | What is your religion?  *Munotevedzera chinamato chipi?* | Traditional  Roman Catholic  Pentecostal  Apostolic Sect  Muslim  None  Other (specify)___________ | 1  2  3  4  5  6 |  |
| Q106a(i) | What is your current occupation?  *Parizvino basa ramunoita nderipi?* | Unemployed  Student  Miner/Mining industry  Farm worker  Professional  Police/Military/Security  Domestic worker  Trucker/Transport business  General worker  Self employed  Vendor  Other (specify)__________ | 1  2  3  4  5  6  7  8  9  10  11 |  |
| Q106a(ii) | What is the current occupation of the head of your household?  *Munhu anotungamira musha uno anoita basa reyi?* | Unemployed  Student  Miner/Mining industry  Farm worker  Professional  Police/Military/Security  Domestic worker  Trucker/Transport business  General worker  Self employed  Vendor  Not applicable  Other (specify)__________ | 1  2  3  4  5  6  7  8  9  10  11  12 |  |
| Q106b | How much is your personal income in a month in US$?  *Unotambira marii pamwedzi?*  **Note**: If no income put ”00”, unsure put “99” | [ ] | US$ |  |
| Q106c | How much is your household income in a month in US$?  *Semhuri ino marii yamunowana pamwedzi imarii?*  **Note:** If no income put “00”, don’t know/unsure put “99” | [ ] | US$ |  |
| Q106d | Do you or any member of your household have a bank account?  *Imi pachezvenyu kana mumwe munhu wemhuri ino mune account ye bhanga****?*** | Yes  No | 1  2 |  |
| Q106e | Are you on medical aid?  *Mune “medical aid’ here?* | Yes  No | 1  2 | If No, skip to Q107a |
| Q106f | If **Yes**, which medical aid are you on?  *Kana ati hongu, ndeipi “medical aid” yaari?* | CIMAS  PSMAS  FML  FLIMAS  CELL MED  HMMAS  ALTFIN  GMB  Generation Health  RailMed  Alliance Health  Corporate 24  Other (specify)____________ | 1  2  3  4  5  6  7  8  9  10  11  12 |  |
|  |  |  |  |  |
|  |  |  |  |  |
| Q107 | What kind of toilet facility does your household usually use?  *Chimbuzi chinoshandiswa nevagari vepano chakamira sei?*    **For community survey ask to observe.** | **Flush or pour flush toilet**  Flush to piped sewer system  Flush with septic tank  Flush to pit latrine  Flush to somewhere else  Flush, don’t know where  **Pit latrine**  Ventilated improved pit latrine  Pit latrine with slab  Pit latrine without slap/open pit  Bucket toilet  No facility/bush/field  Other (specify)_______________ | 1  2  3  4  5  6  7  8  9  10 |  |
| Q108 | What type of fuel does your household mainly use for cooking?  *Nguva zhinji nderipi samba ramuno shandisa kubika mumba muno?* | Electricity  LP gas  Natural gas  Biogas  Paraffin/kerosene  Coal,lignite  Charcoal  Wood  Straw/shrubs/grass  Animal dung  No food cooked in household  Other (specify)____________________ | 1  2  3  4  5  6  7  8  9  10 |  |
|  |  |  |  |  |
|  |  |  |  |  |
|  |  |  |  |  |
|  |  |  |  |  |
| Q109 | Does your household have:  *Munotevedzera chinamato chipi?*  **Read list to respondent** | Electricity  Radio  Television  Non-mobile telephone  Computer  Refrigerator  Battery/generator  Solar panel  Dish/decoder  Washing machine  Borehole  Watch  Mobile phone  Motorcycle/scooter  Car/truck  Boat with motor | No Yes  0 1  0 1  0 1  0 1  0 1  0 1  0 1  0 1  0 1  0 1  0 1  0 1  0 1  0 1  0 1  0 1 |  |
| Q110 | What is the main material of the **floor** of the main house of your household?  *Zvii zvakashandiswa mukugadzira pasi remba ino?*  **Community survey: Observe the main materials** | **Natural floor**  Earth/sand  Dung  **Rudimentary floor**  Wood planks  **Finished floor**  Parquet or polished wood  Vinyl or asphalt strips  Ceramic tiles  Cement  Carpet  Other (specify)__________________ | 1  2  3  4  5  6  7  8 |  |
|  |  |  |  |  |
|  |  |  |  |  |
| Q111 | How often do you watch TV  *Munowona chivhitivhiti kangani?* | Every day  4 to 6 days a week  2 to 3 days a week  Once a week  Never | 1  2  3  4  5  6 |  |
| Q1172 | For women in marriage or co-habiting: How often does your husband or partner watch TV*?*  *Murume wenyu kana kuti wamunogarisana naye anowona chivhitivhiti kangani?* | Every day  4 to 6 days a week  2 to 3 days a week  Once a week  Never  Don’t know | 1  2  3  4  5  6  7 |  |
| Q113 | How often do you read newspapers?  *Munoverenga bepanhau kangani?* | Every day  4 to 6 days a week  2 to 3 days a week  Once a week  Never | 1  2  3  4  5  6 |  |
| Q114 | **For women in marriage or co-habiting:** How often does your husband or partner read the newspapers?  *Murume wenyu kana kuti wamunogarisana naye anoverenga bepanhau kangani?* | Every day  4 to 6 days a week  2 to 3 days a week  Once a week  Never  Don’t know | 1  2  3  4  5  6  7 |  |
| Q115 | How often do you listen to the radio?  *Munoteerera nhepfenyuro(redhiyo) kangani* | Every day  4 to 6 days a week  2 to 3 days a week  Once a week  Never | 1  2  3  4  5  6 |  |
| Q116 | **For women in marriage or co-habiting:** How often does your husband or partner listen to the radio?  *Murume wenyu kana kuti wamunogarisana naye anonoteerera nhepfenyuro(redhiyo) kangani* | Every day  4 to 6 days a week  2 to 3 days a week  Once a week  Never  Don’t know | 1  2  3  4  5  6  7 |  |
| Q117 | Do you have access to the internet on computer or phone?  *Munokwanisa kuwana foni kana kuti kombuyuta?* | Yes  No | 1  2 | If No skip to Q201 |
| Q118 | **For women in marriage or co-habiting:** Does your husband or partner have access to internet on computer or phones?  *Murume wenyu kana kuti wamunogarisana naye anoenda pa “internet” kangani* | Yes  No  Don’t know | 1  2  3 |  |
| Q119 | If **Yes**, how often do you access the internet?  *Kana ati hongu, Unoenda kangani pa “internet”?* | Every day  4 to 6 days a week  2 to 3 days a week  Once a week  Never | 1  2  3  4  5 |  |
| Q120 | **For women in marriage or co-habiting:** If Yes how often does your husband or partner access the internet?  *Murume wenyu kana kuti wamunogarisana naye anoshandisa kangani “internet” ?* | Every day  4 to 6 days a week  2 to 3 days a week  Once a week  Never  Don’t know | 1  2  3  4  5  6 |  |

**SECTION 2: GENERAL KNOWLEDGE ON CERVICAL CANCER**

**In this section I am going to ask you some questions about what you know concerning cervical cancer and please free to let me if you do not know or you are not sure about anything.**

| Q201 | Have you ever heard/seen about messages on cervical cancer?  *Makambonzwa kana kuwona mashoko pamusoro pe gomarara remuromo we chibereko?* | Yes  No | 1  2 | If No skip to Q203 |
| --- | --- | --- | --- | --- |
| Q202 | Where did you hear/see about messages on cervical cancer?  *Makanzwa kupi kana kuwona kupi mashoko pamusoro pe gomarara remuromo wechibereko*  **Multiple selection possible** | Radio  Television  Workplace  Newspaper/magazine  Poster  Billboard  Health/community worker/Counselor  Friends/relatives  Other(Specify) _____ | 1  2  3  4  5  6  7  8 |  |
| Q203 | What are the causes of cervical cancer that you know or have heard about?  *Ndezvipi zvamunoziva kana zvamakambonzwa kuti zvino konzera gomarara remuromo wechibereko****?***  **Multiple selection possible** | Human papilloma virus (HPV)  HIV/AIDS  Don’t know  Other (specify)____________ | 1  2  3 |  |
| Q204 | How is cervical cancer prevented?  *Gomarara remuromo wechibereko rino dzivirirwa sei?*  **Multiple selection possible** | Early screening and treatment  Male circumcision  HPV Vaccination  Stop smoking  Stop drinking alcohol  Eating healthy  Regular exercises  Don’t know  Other (specify)____________ | 1  2  3  4  5  6  7  8 |  |
| Q205a | Do you think that cervical cancer can be treated?  *Mukufunga kwenyu gomarara remuromo wechibereko rino rapika here?* | Yes  No  Don’t know | 1  2  3 |  |
| Q205b | If **Yes**, how do you think it may be treated?  *Kana, Hongu, Munofunga kuti rino rapika sei?*  **Multiple selection possible** | Drugs (Medication)  Surgery  Radiotherapy  Herbs/Traditional medicine  Spiritual means  Don’t know  Other (specify)____________ | 1  2  3  4  5  6 |  |
| Q206 | Have you ever heard about palliative care that is given to cancer patients including those  suffering from cervical cancer?  *Makambonzwa maererano ne kuchengetwa kana rutsigirwo runopiwha vanorwara ne gomarara rechibereko?* | Yes  No | 1  2 | If No skip to Q208 |
| Q207a | If Yes, where did you hear about it?  *Kana ati hongu, makanzwa kupi mashoko aya?*  **Multiple selection possible** | Radio  Television  Workplace  Newspaper/magazine  Poster  Billboard  Health/community worker/Counselor  Friends/relatives  Internet  Other(Specify) _____ | 1  2  3  4  5  6  7  8  9 |  |
| Q207b | Where are palliative care services given in Harare?  *Ndekupi kuno chengetwa kana kutsigirwa vanhu vane gomarara remuromo wechibereko?*  **Multiple selection possible** | Island Hospice  Hospice and Palliative care association of Zimbabwe  Nursing homes  Don’t know  Other (specify)____________ | 1  2  3  4 |  |
| Q208 | Do you think that cervical cancer is somehow associated with HIV/AIDS***?***  *Mukufunga kwenyu mungati gomarara remuromo wechibereko rino kuwadzana ne HIV/AIDS* | Yes  No  Don’t know | 1  2  3 |  |
| Q209 | In your opinion do you think cervical cancer is a problem in Zimbabwe?  *Mukuwona kwenyu munofunga kuti gomarara remuromo wechubereko rave dambudziko munyika ye Zimbabwe* | Yes  No  Don’t know | 1  2  3 |  |

**SECTION 3: ACCESS AND USE OF CERVICAL CANCER TREATMENT AND PALLIATIVE CARE**

**In the next section I am going to ask you questions regarding access and use of cervical cancer treatment and palliative care.**

***Muchikamu chirikutevera ndichakubvubnzai pamuroro pekuwanikwa kwekurapwa nekuchengetwa kwecanhu vane gomara remuromo wechibereko***

| **MEASURE OF ACCESS TO CERVICAL CANCER TREATMENT** | | | | |
| --- | --- | --- | --- | --- |
| **HEALTHY WOMEN** | | | | |
| Q301 | Do you know where to get or to refer someone to get cervical cancer treatment and palliation services?  *Munoziva kwekuwana kana kwekureva kuti mhunu ano ongororwa kuti ane kana hana gomarara remuromo wechibereko uye nzvimbo dzinobatsira avo vane gomarara?* | Yes  No | 1  2 | If No skip to Q303 |
| Q302a | Generally, where would you go or refer someone for cervical cancer treatment services?  *Munga ende kupi kana kureva kuti mumwe anowana rubatsiro rune chekuita nezve gomarara remuromo wechibereko*  **Multiple responses possible** | Clinic  Hospital  Private Practitioner  New Start Centre  Traditional healer  Prophet or pastor  Other (specify)____________ | 1  2  3  4  5  6 |  |
| Q302b | Generally, where would you go or refer someone for cervical cancer palliative care services?  *Munga ende kupi kana kureva kuti mumwe anowana rubatsiro rune chekuita nekuchengetwa kwe vane gomarara remuromo wechibereko*  **Multiple responses possible** | Island Hospice  Hospice and Palliative care association of Zimbabwe  Nursing homes  Don’t know  Other (specify)__________ | 1  2  3  4 |  |
| Q303 | How far is your nearest health facility from your home?  *Kiriniki kana chipatara chamungati chiripa duze nemusha wenyu chiri kure zvakadii?*  **Probe for an estimate in km** | [ ] | Km |  |
| Q304 | How do you usually get to your nearest health facility?  *Munoends sei kukirinki/chipatara****?*** | Walking  Public transport (e.g commuter omnibus or bus)  Private car  Motor cycle  Other (specify)____________ | 1  2  3  4 |  |
| Q305a | How long does it take you to get there?  *Zvinokutorerai nguva yaka reba sei kuti musvike ikoko?* | [ ] | Minutes |  |
| Q305b | Do you afford the fees charged by your local health facility?  *Munokwanisa kubhadara mari dzinoda kukokwa kukiriniki/chipatara?* | Yes  No | 1  2 |  |
| Q305c | How much does your health facility charge for a single visit?  *Panguva yoga yamunoshanyira kiriniki yenyu munobhadara marii ?* | [ ] | US$ |  |
| Q306a | In your opinion do you think that your local health facility has the capacity to treat cervical cancer?  *Mukuwona kwenyu mungati kirniki yenyu inogona kurapa gomarara remuromo wechibereko* | Yes  No  Don’t know |  |  |
| Q306b | If No or DNK, where do you think people can be treated of cervical cancer in Harare?  *Kana ati kwete kana kuti hazive-*  *Munofunga kuti vanhu vanga ende kupi ku Harare kuno rapwa gomarara remuromo wechibereko?*  **Multiple responses possible** | Harare Hospital  Parirenyatwa Hospital  Private hospitals  Private doctors  Traditional healers  Prophets and pastors  Other (specify)__________ | 1  2  3  4  5  6 | If Yes to Q306a skip to Q306c |
| Q306c | If you were to be diagnosed of cervical cancer today do you think you would have access to treatment and palliative care services in Harare?  *Kurikunzi nhasi mabatwa kuti munegomarara remuromo wechibereko munofunga kuti mungawane kupirubatsiro kana kurapwa mu Harare?* | Yes  No | 1  2 |  |
| Q306d | Where would you go to seek treatment?  *Munga ende kupi kuno rapwa?*  **Multiple response possible** | Harare Hospital  Parirenyatwa Hospital  Local health facility  Private hospitals  Private doctors  Traditional healers  Prophets and pastors  Other (specify)__________ | 1  2  3  4  5  6  7 |  |
| Q306e | Where would you go to seek for palliative care services?  *Munga ende kupi kuno wana rubatsiro kune avo vanochengeta vane gomarara?*  **Multiple responses possible** | Island Hospice  Hospice and Palliative care association of Zimbabwe  Nursing homes  Don’t know  Other (specify)__________ | 1  2  3  4 |  |
| Q307a | Do you think people with cervical cancer are able to access treatment and palliative care services in Harare?  *Munofunga kuti vanhu vanga ende kupi ku Harare kuno rapwa gomarara remuromo wechibereko kana kuno wana rubatsiro kune vanochengeta vano rwara negomarara?* | Yes  No  Don’t know | 1  2  3 |  |
| Q307b | Do you think that Harare has adequate specialists to treat and manage cervical cancer patients?  *Munofunga kuti mu Harare mune anamazvikokota kavakwana uye vachikwanisa kurapa vese avo vane gomarara remuromo wechibereko?* | Yes  No | 1  2 |  |
| Q308 | What challenges do you think cervical cancer patients potentially face in trying to access treatment and palliative care services in Harare?  *Munofunga kuti ndeapi matambudziko angasangane neavo vanegomarara remuromo wechibereko kana vachida kuno rapwa kana kuti kunowana rubatsiro kune avo vano chengeta vanegomarara?*  **Read out list to respondent**  **Multiple selection possible** | Lack of transport to go the health centres.  Lack of finances to pay for the services.  Few centres offer the specialized services.  Bad attitude of health professionals  Stigma from the society  Stock-outs of drugs (medication) at health facilities.  Bureaucratic processes in accessing treatment services  Lack or dysfunctional equipment at health centres.  Other (specify)____________ | 1  2  3  4  5  6  7  8 |  |
| **CERVICAL CANCER SURVIVORS** | | | | |
| Q309 | What made you go for cervical cancer screening just before your diagnosis?  *Chii chakaita kuti muno ongororwa imi musati mabatwa kuti munegomarara remuromo wechibereko?*  **Multiple selection possible** | Routine screening  Friend/relative advice  Health problem  Health professional’s advice  Other (specify)____________ | 1  2  3  4 |  |
| Q311a | Where were you first screened and suspected of cervical cancer?  *Ndekupi kwamakatanga kuongororwa neku fungidzirwa kuti mune gomarara remuromo wechibereko?* | Clinic  Hospital  Private Practitioner  New Start Centre  Other (specify)____________ | 1  2  3  4 |  |
| Q311b | What is the exact name of the health facility or practitioner?  *Zita chairochairo rechipatara/kiriniki kanadokota akaku udzai kuti mune gomarara remuromo wechibereko rinotii?* | [ ] |  | If not sure put “Not sure” |
| Q311c | Where were you referred for further investigations (histological tests) to confirm your diagnosis?  *Makanzi muende kupi kuno tarisiswa kusimbisisa kuti mune gomarara remuromo wechibereko?* | Harare Hospital  Parirenyatwa Hospital  Private laboratories  Private hospital/ Dr’s surgery  Other (specify)__________ | 1  2  3  4 |  |
| Q312 | How far is your home from the health facility you were screened and suspected of cervical cancer?  *Kure sei ku kuriniki/chipatara chamakano udzwa pekutanga kana kufungidzirwa kuti mune gomarara remuromo wechibereko?*  **Probe for an estimate in km** | [ ] | km |  |
| Q313 | How did you go to the health facility where you were first screened and suspected of cervical cancer?  *Makaenda neyi kuriniki/chipatara pamaka udzwa pekutanga kana kufungidzirwa kuti mune gomarara remuromo wechibereko?* | Walking  Public transport (e.g commuter omnibus or bus)  Private car  Motor cycle  Other (specify)____________ | 1  2  3  4  5 |  |
| Q314 | How long did it take you to get to the health facility?  *Zvakatora nguva yakareba sei kuti musvike kukiriniki/chipatara?* | [ ] | Minutes |  |
| Q315 | How much money were you asked to pay for screening services?  *Makanzi mubhadare marii kuti muongororwe?* | [ ] | US$ | Put 0000 if no charge, if on medical aid covered put 9999 |
| Q316 | **If charged:** Was the amount affordable to you?  *Makakwanisa kubhadara mari dzai diwa?* | Yes  No | 1  2 | Skip if answer to Q315 is 0000 or 9999 |
| Q317 | How many times were you screened for cervical cancer in your life?  *Muhupenyu wenyu wese makano ongororwa kangani gomarara remuromo wechibereko?* | [ ] | Put 9999 if cannot remember |  |
| Q318a | Do you have access to treatment for your condition?  *Munokwanisa kurapwa zvamurikurwara nazvo izvezvi?* | Yes  No | 1  2 |  |
| Q318b | Do you have access to specialist services for your condition?  *Munokwanisa kunowna anamazvikokota mukurapa gomorara remuromo wechibereko?* | Yes  No | 1  2 |  |
| Q318c | How many times have you been seen by a specialist in the last 3 months  *Mumwedzi mitatu yapfuura makawonekwa kangani nana mazvikokota?* | [ ] |  | Put 00 if not seen. |
| Q319 | How long did it take for you to be put on treatment from the time of diagnosis?  *Zvakatora nguva yareba sei kuti muzoiswa pamishonga shure kwekunge maudzwa kut mune gomarara remuromo wechibereko?* | Months Days  [ ] [ ] | Put 9999 if not yet on treatment |  |
| Q320 | Where were you commenced on treatment?  *Matanga kurapwa murikupi?* | Harare Hospital  Parirenyatwa Hospital  Private nursing home  Private hospital/ Dr’s surgery  Not yet on treatment  Other (specify)__________ | 1  2  3  4  5 |  |
| Q321a | What kind of treatment were you put on?  *Makarapwa nenzira ipi?*  **Multiple selection possible** | Drugs (Medication)  Surgery  Radiotherapy  Other (specify)____________ | 1  2  3  4 |  |
| Q321b | Are you receiving palliative care services?  *Murikwana rubatsiro runopiwa neavo vanochengeta vano rwara ne gomarara* | Yes  No | 1  2 | If No skip to Q322a |
| Q321c | If **Yes,** where are you getting the services?  *Murikuri wana kurpi rubatsiro uru?* | Island Hospice  Hospice and Palliative care association of Zimbabwe  Nursing homes  Home  Other (specify)__________ | 1  2  3  4 |  |
| Q321d | What other medication or services have you received for your condition apart from those provided at health facility?  *Ndeipi mishonga kana rubatsiro rwamawana rusiri rwe kuchipatara?*  **Multiple selection possible**  **Probe for more**. | Herbs/traditional medicine  Spiritual materials (water, oil, or stones)  Prayers  Other (specify)____________ | 1  2  3 |  |
| Q322a | How much have you paid or are you paying on average for your treatment in one month?  *Mumwedzi mumwe chete mabhadara marii kana kuti mri kubhadara narii kuti murapwe?*  **Probe for estimates** | [ ] | US$/per month | If no payment put 0000 and skip to Q323 |
| Q322b | How much have you paid or are you paying on average for your palliative care services in one month?  *Mabhadara marii kana kuti muri kubhadara marii pamwedzi kuti muane rubatsiro rokuchengetwa kweavo vane gomarara?*  **Probe for estimates** | [ ] | US$/per month | If no payment put 0000 and skip to Q323 |
| Q322b | Are (Were) these costs affordable to you or your family?  *Mari dzinodiwa kuti murapwe munodzikwanisa kana kuti mhuri yenyu inodzikwanisa here?* | Yes  No | 1  2 |  |
| Q323a | What challenges did you or do you face to access treatment services?  *Ndeapi matambudziko amunosangana nawo kana muchino rapwa kukiriniki/chipatara?*  **Read out list to respondent**  **Multiple selection possible** | Lack of transport to go the health centres.  Lack of finances to pay for the services.  Few centres offer the specialized services.  Bad attitude of health professionals  Stigma from the society  Stock-outs of drugs (medication) at health facilities.  Bureaucratic processes in accessing treatment services  Lack or dysfunctional equipment at health centres.  Other (specify)____________ | 1  2  3  4  5  6  7  8 |  |
| Q323b | Have you been satisfied with the services that you have received in this health facility?  *Murikufara nekurapwa kwamuri kuitwa kukiriniki/chipatara ichi?* | Yes  No | 1  2 |  |
| Q323c | Would you continue to come back for treatment services in this facility even if you were given other choices?  *Mungadzoke here kuzorawa kurikiniki iyi kana mukapiwa mukana wekusarudza kwekuno rapwa?* | Yes  No | 1  2 |  |
| Q323d | Given your experiences so far would you recommend a friend or relative to receive treatment or palliative care in this health facility?  *Muchitarisa zvamasangana nazvo munga kurudzire shamwari kana hama yenyu kuti inorapwa kana kunwa rubastiro runopiwa vane gomarara kukiriniki kana chipatara ichi*? | Yes  No | 1  2 |  |
| **UTILIZATION OF CERVICAL CANCER TREATMENT** | | | | |
| **HEALTHY WOMEN** | | | | |
| Q324a | How many times did you visit your health facility or doctor in the last 6 months?  *Mumwedzi nhanhatu yapfuura waenda kangani kukiriniki kana kunowonekwa na chiremba?* | [ ] |  | If none put 0000 |
| Q324b | Do you have a regular doctor whom you see when you require health services?  *Munachiremba wamunno wanzo enda kuno wona kana marwara?* | Yes  No | 1  2 |  |
| Q325 | If you are not feeling well where would you go **first**?  *Kana musiri kunza zvakanaka ndekupi kwamuno tanga kuyenda kuno rapwa*? | Clinic  Hospital  Private Practitioner (Doctor)  New Start Centre  Traditional healer  Prophet or pastor  Other (specify)____________ | 1  2  3  4  5  6 |  |
| Q326 | If you were to be given some medication or treatment for a disease would adhere to it?  *Kurikunzi mapihwa mishonga kana kuraphwa chero chirhwere munogona kuteedzera zvinenge zvichidiwa?* | Yes  No | 1  2 |  |
| Q327 | Have you ever been screened for cervical cancer?  *Makambo ongororwa pachitariswa huvepo hwegomara romuromo wechibereko?* | Yes  No | 1  2  3 | If No skip to Q332a |
| Q328 | When you were last screened?  *Makapedzesera rini kuongororwa?* | Month Year  [ ] [ ] |  | If not known put 9999 |
| Q329 | Where were you screened?  *Makawongororwa kupi?* | Harare Hospital  Parirenyatwa Hospital  New Start Centre  Private hospital/Dr’s surgery  Cancer Association  Other (specify)___________ | 1  2  3  4  5 |  |
| Q330 | How much where you asked to pay for the screening services?  *Makanzi mubhadare mariikuti muongororwe?* | [ ] | US$ | If nothing put 0000 and if medical aid covered put 9999 |
| Q331 | Were the charges affordable to you or your household?  *Mari dzinodiwa kuti murapwe munodzikwanisa kana kuti mhuri yenyu inodzikwanisa here?* | Yes  No | 1  2 |  |
| Q332a | Who do you believe can manage cervical cancer better?  *Ndiyani wamunofunga kuti anogona kurapa gomarara remuromo wechibereko zviri nani?* | Health professionals  Traditional healers  Prophets and pastors  Other (specify)____________ | 1  2  3 |  |
| Q332b  ‘ | What challenges do you usually face in using health services?  *Ndeapi matambudziko amunosangana nawo kana muchino rapwa kukiriniki/chipatara?*  **Read out list to respondent**  **Multiple selection possible** | Lack of transport to go the health centres.  Lack of finances to pay for the services.  Bad attitude of health professionals  Stigma from the society  Stock-outs of drugs (medication) at health facilities.  Bureaucratic processes in accessing treatment services  Lack or dysfunctional equipment at health centres.  Poor quality of care  Side effect of medication or treatments  Other (specify)____________ | 1  2  3  4  5  6  7  8  9 |  |
| **CERVICAL CANCER SURVIVORS** | | | | |
| Q333a | How many times have you visited your health facility or doctor for treatment/check up in the last 6 months?  *Mumwedzi nhanhatu yapfuura maenda kangani kukiriniki kana kunachiremba kunorapwa kana kutariswa?* | [ ] |  |  |
| Q333b | Do you have a regular doctor whom you see when you require health services?  *Munachiremba wamunowona kana muchida kurapwa?* | Yes  No |  |  |
| Q334a | What treatment are you on or have you received for your condition?  *Makarapwa nenzira ipi kana kuti murikurapwa nenzira yipi?*  **Multiple selection possible** | Drugs (Medication)  Surgery  Radiotherapy  Herbs/Traditional medicine  Spiritual means  Other (specify)____________ | 1  2  3  4  5 |  |
| Q334b | Where do you or did you get treatment for your condition?  *Munoenda kupi kuno rapwa chirwere chamunacho?*  **Multiple selection possible** | Harare Hospital  Parirenyatwa Hospital  Local Clinic  Private hospital/Dr’s surgery  Private nursing home  Other (specify)___________ | 1  2  3  4  5 |  |
| Q335 | How much are you paying or were you asked to pay for your treatment?  *Muri kubhadara kana kuti makabhadara marii kuti murapwe?* | [ ] | US$ | If nothing put 0000 and if medical aid covers put 9999 |
| Q336 | Are (Were) these fees affordable to you or your household?  *Mari dzinodiwa kuti murapwe munodzikwanisa kana kuti mhuri yenyu inodzikwanisa here*? | Yes  No | 1  2 | Skip if answer to Q335 is 0000 or 9999 |
| Q337 | Who do you believe can manage cervical cancer better?  *Ndiyani wamunofunga kuti anogona kurapa gomarara remuromo wechibereko zviri nani?* | Health professionals  Traditional healers  Prophets and pastors  No one  Other (specify)____________ | 1  2  3  4 |  |
| Q338 | What challenges do you usually face in using health services?  *Ndeapi matambudziko amunosangana nawo kana muchino rapwa kukiriniki/chipatara?*  **Read out list to respondent**  **Multiple selection possible** | Lack of transport to go the health centres.  Lack of finances to pay for the services.  Few centres offer the specialized services.  Bad attitude of health professionals  Stigma from the society  Stock-outs of drugs (medication) at health facilities.  Bureaucratic processes in accessing treatment services  Lack or dysfunctional equipment at health centres.  Side-effects from drugs or treatment  Other (specify)____________ | 1  2  3  4  5  6  7  8  9 |  |
| **I am now going to ask you questions regarding your perceptions on cervical cancer treatment and palliation services. Please tell me if you ‘agree’ or disagree. Note: Probe if they “agree strongly” or “agree somewhat”, and if they “disagree strongly” or “disagree somewhat”.**  *Ikozvino ndave kuda kukubvunzai mibvunzo maererano nemaonero enyu erubatsiro rapwa kwegomarara remurmo wechibereko nekuchengetwa kwavanoitwa. Ndinokumbira kuti mundiudze kana muchibvuma kana kuramba* | | | | |

|  | **SELF EFFICACY** | Strongly  Agree | Agree | Neither agree or disagree | Disagree | Strongly disagree |
| --- | --- | --- | --- | --- | --- | --- |
| Q339 | I can discuss experiences of cervical cancer with other women.  *Ndino kwanisa kutaura nevamwe vanhukadzi pamusoro pezvandaka sangana nazvo zvakanangana negomarara remuromo wechibereko* | 5 | 4 | 3 | 2 | 1 |
| Q340 | I can discuss experiences of cervical cancer with my family members.  *Ndino kwanisa kutaura neve mumhuri mangu pamusoro pezvandaka sangana nazvo zvakanangana negomarara remuromo wechibereko* | 5 | 4 | 3 | 2 | 1 |
| Q341 | I can discuss experiences of cervical cancer with my friends.  *Ndino kwanisa kutaura neshamwari dzangu pamusoro pezvandaka sangana nazvo zvakanangana negomarara remuromo wechibereko* | 5 | 4 | 3 | 2 | 1 |
| Q342 | I am afraid to discuss experiences of cervical cancer with anyone [R].  *Ndinotya kutaura nevamwe pamusoro pe zvandaka sangana nazvo zvakanangana negomarara remuromo wechibereko* | 5 | 4 | 3 | 2 | 1 |
|  | **AVAILABILITY** | 5 | 4 | 3 | 2 | 1 |
| Q343 | Health workers at the hospital/clinic are sensitive to cervical cancer patients.  *Vashandi vemuchipatara/kiriniki vanonzwisisa vanu vano rwara regomarara romuromo wechibereko* | 5 | 4 | 3 | 2 | 1 |
| Q344 | The hospital/clinic in my community offers timely services for people with cervical cancer.  *Chipatara/kiriniki chinopa rubatsiro nekuchimbidza kune avo vano rwara regomarara romuromo wechibereko* | 5 | 4 | 3 | 2 | 1 |
| Q345 | The hospital staff can handle cervical cancer cases with confidentiality.  *Vashandi vemuchipatara/kiriniki vanochengetedza kurwara kwe avo vano rwara negomarara romuromo wechibereko zvakavanzika* | 5 | 4 | 3 | 2 | 1 |
| Q346 | Awareness of cervical cancer is done in my community.  *Kuzivisa vanhu pamusoro pegomarara remuromo wechibereko kunoitwa munharaunda yangu* | 5 | 4 | 3 | 2 | 1 |
| Q347 | The local hospital offers cervical cancer screening to women  *Chipatara chemnharudna ino chino ongorora vanhukadzi gomarara remuromo wechibereko mahara* | 5 | 4 | 3 | 2 | 1 |
| Q347 | The local hospital offers cervical cancer vaccination to young girls  *Chipatara chemu nharaunda ino chinopa vasikana vechidiki mushonga wekudzivirira gomarara remuromo wechibereko* | 5 | 4 | 3 | 2 | 1 |
| Q348 | The local hospital offers treatment to women with cervical cancer.  *Chipatara chemu nharaunda ino chinorapa vanhukadzi gomarara remuromo* wechibereko | 5 | 4 | 3 | 2 | 1 |
| Q349 | The local hospital offers palliative care to cervical cancer patients.  *Chipatara chemu nharaunda ino chinopa rubastiro rwekuchengeta avo vane gomarara remuromo wechibereko* | 5 | 4 | 3 | 2 | 1 |
| Q350 | The local hospital offers health education about cervical cancer to women.  *Chipatara chemunharaunda ino chinopa dzidziso pamusoro pegomarara remuromo wechibereko* | 5 | 4 | 3 | 2 | 1 |
| Q351 | The local hospital offers laboratory investigations for women suspected of cervical cancer.  *Chipatara chemunharaunda ino chinopa rubatsiro rweku ongorora mumalebhu kunevanhukadzi vvarikufungidzirwa kuti vane* *negomarara remuromo wechibereko* | 5 | 4 | 3 | 2 | 1 |
| Q352 | The local hospital has adequate equipment for treatment of cervical cancer.  *Chipatara chemunharaunda ino chine mishina yekurapa vese vano rwara negomarara remuromo wechibereko* | 5 | 4 | 3 | 2 | 1 |
| Q353 | The local hospital has adequate trained staff to provide cervical cancer treatment.  *Chipatara chirimunharaunda ino chine vashandi vakadzidziswa kurapa avo vane gomarara remuromo* wechibereko | 5 | 4 | 3 | 2 | 1 |
| Q354 | The local hospital offers counselling to cervical cancer patients and their partners/families.  *Chipatara chemunharaunda ino chinopa mazanno ukurarama kune vanegomarara remuromo wechibereko* | 5 | 4 | 3 | 2 | 1 |
| Q355 | The local hospital offers treatment to **all** cervical cancer patients in this community.  *Chipatara chemunharaunda ino chinorapa vese vano rwara negomarara remuromo wechibereko* | 5 | 4 | 3 | 2 | 1 |
| Q356 | Churches in my community provide support to people/families with cervical cancer.  *Machechi emunharaunda ino anopa rubatsiro kunavhu/nemhuri dzine gomarara remuromo wechibereko* | 5 | 4 | 3 | 2 | 1 |
| Q357 | Traditional healers provide cervical cancer treatment in this community  *N’anga dzemunharaunda ino dzino rapa gomarara remuromo wechibereko* |  |  |  |  |  |
| Q358 | Medical services for survivors of cervical cancer are not available in my community[R]  *Hapana zvirongwa zvekurapwa kwevanhu vararama shure kwekuva negomarara remuromo wechibereko* | 5 | 4 | 3 | 2 | 1 |
|  | **AFFORDABILITY** |  |  |  |  |  |
| Q359 | Cervical cancer treatment services are affordable in my community  *Kurapwa gomarara remuromo wechibereko akudhuri.* | 5 | 4 | 3 | 2 | 1 |
| Q360 | Most people in my community are able to pay the local clinic/hospital fees for health services.  *Vanhu vazhinji vemunharaunda yangu vanokwanisa kubhadara mari dzino diwa kukiriniki /chipatara kuti varapwe.* | 5 | 4 | 3 | 2 | 1 |
| Q361 | Hospitals/clinics in my community offer screening for cervical cancer for free.  *Zvipatara/ makiriniki emunharaunda yangu zvinovheneka mahara.* | 5 | 4 | 3 | 2 | 1 |
| Q362 | Hospitals/clinics in my community offer for cervical cancer treatment services for free.  *Zvipatara/ makiriniki emunharaunda yangu zvinorapa mahara.* | 5 | 4 | 3 | 2 | 1 |
| Q363 | Hospitals/clinics in my community offer free treatment services for cervical cancer patients who cannot afford to pay.  *Zvipatara/ makiriniki emunharaunda yangu zvinorapa mahara avo vasinga kwanise kubhadara.* | 5 | 4 | 3 | 2 | 1 |
| Q364 | Cervical cancer treatment is cheaper abroad than in my local hospital/clinic.  *Kurapwa gomarara remuromo wechibereko kwakachipha kunze kwenyika tichienzanisa nekurapwa muno* | 5 | 4 | 3 | 2 | 1 |
| Q365 | I get better value for money for cervical cancer treatment abroad than in my local hospital/clinic.  *Ndiwana rubatsiro rwakakwana kunze kwenyika kupfura muzvipatara zvenumuno, kuburikidza nemari yandinenge ndabhadara* | 5 | 4 | 3 | 2 | 1 |
|  | **SOCIAL SUPPORT** |  |  |  |  |  |
| Q366 | My partner/husband [would] supports me to go for cervical cancer screening.  *Murume/mumwe wangu anondikurudzira kuti ndiyende kuno ongororwa gomarara remuromo* wechibereko | 5 | 4 | 3 | 2 | 1 |
| Q367 | My partner/husband [would] supports me to go for cervical cancer treatment  *Murume/mumwe wangu anondikurudzira kuti ndiyende kuno rapwa gomarara remuromo wechibereko* | 5 | 4 | 3 | 2 | 1 |
| Q368 | My friends supports me to go for cervical cancer treatment.  *Shamwari dzangu dzinondikurudzira kuti ndiyende kuno rapwa gomarara remuromo wechibereko* | 5 | 4 | 3 | 2 | 1 |
| Q369 | My family supports me to go for cervical cancer treatment  *Mhuri yangu inondikurudzira kuti ndiyende kuno rapwa gomarara remuromo wechibereko* | 5 | 4 | 3 | 2 | 1 |
| Q370 | Cervical cancer treatment is acceptable in my community  *Kurapwa gomarara remuromo wechibereko chinhu chakanguchirika munharaunda yang*u | 5 | 4 | 3 | 2 | 1 |
| Q371 | Leaders in my community encourage women to be screened and treated for cervical cancer.  *Vatungamiri venharaunda inovano kurudzira kut vakadzi vaende kuno ongororwa nekurapwa gomarara remuromo wechibereko* | 5 | 4 | 3 | 2 | 1 |
| Q372 | My community believes that cervical cancer can be treated.  *Vanhu vemunharaunda yangu vanotenda kuti gomarara remuromo wechibereko rino rapika* | 5 | 4 | 3 | 2 | 1 |
| Q373 | My community does not stigmatize women with cervical cancer.  *Vanhu vemu nharaunda yangu*  *Havasarudze vanhukadzi vane gomarara remuromo wechibereko* | 5 | 4 | 3 | 2 | 1 |
|  | **ATTITUDES** | 5 | 4 | 3 | 2 | 1 |
| Q374 | Cervical cancer patients should not be stigmatized  *Vano rwara negomarara remuromo wechibereko havafanirwe kusarudzwa* | 5 | 4 | 3 | 2 | 1 |
| Q375 | I encourage others to be screened and treated for cervical cancer  *Ndinokurudzira vamwe kuti vaende kuno ongororwa nekurapwa gomarara remuromo wechibereko*. | 5 | 4 | 3 | 2 | 1 |
| Q376 | I am afraid of cervical cancer treatment [R]  *Ndotya kuenda kunorapwa gomarara remuromo wechibereko* | 5 | 4 | 3 | 2 | 1 |
| Q377 | Getting results of cervical cancer screening is scary [R]  *Kuwana zvabuda mukuongororwa huvepo wegomarara remuromo wechibereko kunotyisa* | 5 | 4 | 3 | 2 | 1 |
| Q378 | Women should go for cervical cancer screening only when they experience serious health problems [R]  *Vanhukadzi vanofanira kuyenda kuno ongororwa chete kana hutano wavo usisina kumira* ***zvakanaka.*** | 5 | 4 | 3 | 2 | 1 |
| Q379 | Cervical cancer treatment is for people with money [R]  *Kunorapwa gomarara remuromo wechibereko ndekwe vanhu vane mari* | 5 | 4 | 3 | 2 | 1 |
| Q380 | Cervical cancer screening is for promiscuous people [R]  *Kuongororwa huvepo hwegomarara remuromo wechibereko kunoitwa nevanhu vasina kuzvibata* | 5 | 4 | 3 | 2 | 1 |
| Q381 | I am too busy to go for cervical cancer treatment [R]  *Ndine zvakawanda zvakanyanya kuti ndiyende kuno rapwa gomarara remuromo wechibereko* | 5 | 4 | 3 | 2 | 1 |
| Q382 | I do not have time to go for cervical cancer treatment [R]  *Andina nguva yekuyenda kuno rapwa gomarara remuromo wechibereko* | 5 | 4 | 3 | 2 | 1 |
| Q383 | Cervical cancer treatment procedure is embarrassing [R]  *Zvinonyadzisa kunorapwa gomarara remuromo wechibereko* | 5 | 4 | 3 | 2 | 1 |
|  | **BELIEFS** |  |  |  |  |  |
| Q384 | Screening is important for early treatment of cervical cancer  *Zvakakosha kuyenda pachinenguva kuno ongororwa huvepo we gomarara remuromo wechibereko.* | 5 | 4 | 3 | 2 | 1 |
| Q385 | Cervical cancer treatment saves lives.  *Kurapwa gomarara remuromo wechibereko zvino chengetedza hupenyu* | 5 | 4 | 3 | 2 | 1 |
| Q386 | Cervical cancer treatment gives a woman and their family peace of mind.  *Kurapwa gomarara remuromo wechibereko zvinopa munhukadzi nemhuri yake zororo mupfugwa* | 5 | 4 | 3 | 2 | 1 |
| Q387 | Cervical cancer treatment gives a woman control over her health.  *Kurapwa gomarara remuromo wechibereko zvinopa munhukadzi samba pamusoro pehutanho hwake*. | 5 | 4 | 3 | 2 | 1 |
| Q388 | HIV testing is optional when being screened for cervical cancer  *Kuongororwa HIV hakumanikidzwe kana uchiongororwa huvepo hwe gomarara remuromo wechibereko* | 5 | 4 | 3 | 2 | 1 |
| Q389 | Cervical cancer treatment is not painful  *Kurapwa gomarara remuromo wechibereko kunorwadza* | 5 | 4 | 3 | 2 | 1 |
| Q390 | Cervical cancer treatment has no side-effects  *Kurapwa gomarara remuromo wechibereko hakuna zvakuno kanganisa* | 5 | 4 | 3 | 2 | 1 |
| Q391 | Cervical cancer treatment is for all women regardless of background  *Kurapwa gomarara remuromo wechibereko ndekwe munhu wese zvisineyi kwaanobva* | 5 | 4 | 3 | 2 | 1 |
| Q392 | Cervical cancer cannot be treated [R]  *Gomarara remuromo wechibereko harirapike* | 5 | 4 | 3 | 2 | 1 |
| Q393 | Cervical cancer patients do not survive long even when treated [R]  *Vane gomarara remuromo wechibereko havararame kunyagwe varapwa.* | 5 | 4 | 3 | 2 | 1 |
| Q394 | Cervical cancer is best treated with herbs/traditional medicines.  *Gomarara remuromo wechibereko rino rapika zvakanaka ne mishonga yechi vanhu* | 5 | 4 | 3 | 2 | 1 |
| Q395 | Cervical cancer is best treated using spiritual means performed by prophets and pastors.  *Gomarara remuromo wechibereko rino rapwa zvirinani nevezve Mweya- Maporofita namafundisi* | 5 | 4 | 3 | 2 | 1 |
| Q396 | Cervical cancer treatment is best done abroad.  *Zvakanaka zvikuru kurapwa gomarara remuromo wechibereko kunze kwenyika* | 5 | 4 | 3 | 2 | 1 |
| Q397 | Health professionals abroad provide better care for cervical cancer patients.  *Madokota arikunze kwenyika vano rapa zviri nani avo vane gomarara remuromo wechibereko* | 5 | 4 | 3 | 2 | 1 |
| Q398 | Cervical cancer patients treated abroad have better survival chances.  *Vane gomarara remuromo wechibereko vane mukana urinani wekuti vararame* | 5 | 4 | 3 | 2 | 1 |
|  | **LOCUS OF CONTROL** |  |  |  |  |  |
| Q399 | I am responsible for my health  *Ndini ndino fanira kuchengetedza hutano wangu* | 5 | 4 | 3 | 2 | 1 |
| Q400 | No one needs to know if I am going for cervical cancer treatment  *Hapana anofanira kuziva kuti ndiri kuenda kuno ongororwa huvepo we gomarara remuromo wechibereko* | 5 | 4 | 3 | 2 | 1 |
| Q401 | I should not be ignorant of my status relating to cervical cancer  *Andifanirwe kusaziva chimiro changu zvakanangana ne gomarara remuromo wechibereko* | 5 | 4 | 3 | 2 | 1 |
|  | **THREAT** |  |  |  |  |  |
| Q402 | Cervical cancer is painful [R]  *Gomarara remuromo wechibereko inorwadza* | 5 | 4 | 3 | 2 | 1 |
| Q403 | Cervical cancer is smelly [R]  *Gomarara remuromo wechibereko inonhuhwa* | 5 | 4 | 3 | 2 | 1 |
| Q404 | Cervical cancer can cause death [R]  *Gomarara remuromo wechibereko inowuraya* | 5 | 4 | 3 | 2 | 1 |
|  | **QUALITY OF CARE** |  |  |  |  |  |
| Q405 | Test results for cervical cancer screening are immediate  *Zvinenge zvabuda mukutariswa pachitsvagwa huvepo hwe gomarara remuromo wechibereko zvinobuda ipapo ipapo* | 5 | 4 | 3 | 2 | 1 |
| Q406 | Cervical cancer screening does not take too long  *Hazvitori nguva yakarebaka Kuongororwa pachitsvagwa huvepo hwegomarara remuromo wechibereko* | 5 | 4 | 3 | 2 | 1 |
| Q407 | Health care workers who perform cervical cancer treatment are well trained  *Vashandi vanorapa gomarara remuromo wechibereko vakadzidziswa zvemhando* *yepamusoro* | 5 | 4 | 3 | 2 | 1 |
| Q408 | Health care workers who perform cervical cancer treatment are very helpful  *Vashandi vanorapa gomarara remuromo wechibereko vanobatsira zvikuru* | 5 | 4 | 3 | 2 | 1 |

**Interviewer’s observations**

**____________________________________________________________________________________________________________________________________________________________________________________________________________________________________________________________**

**Comments on specific questions**

**____________________________________________________________________________________________________________________________________________________________________________________________________________________________________________________________**

**Any other comments**

**____________________________________________________________________________________________________________________________________________________________________________________________________________________________________________________________**

**Remarks:** Thank the participant for their time and proceed to the next respondent.

**----------------------------------------------------------------THE END---------------------------------------------------------------**
