# Supplementary material for: Health system constraints affecting treatment and care among women with cervical cancer in Harare, Zimbabwe
Source: BMC Health Serv Res. 2019 Nov 12;19:829. doi: 10.1186/s12913-019-4697-6 (PMC6852958; doi:10.1186/s12913-019-4697-6)
Supplement: Supplementary file 2 — Additional file 2. Validated structured questionnaire for health workers [English]. [file 12913_2019_4697_MOESM2_ESM.docx]

**HEALTH WORKER SURVEY**

**Study title: Equity in access and utilization of cervical cancer treatment and palliation services in Harare, Zimbabwe.**

**SPEAK TO THE HEAD OF THE HOUSEHOLD:** Hello. My name is Oscar Tapera and I am a PhD student at the University of Pretoria, South Africa. We are interviewing health workers at [name of health facility] in order to obtain your views, opinions and experiences on cervical cancer treatment and palliative care services in Harare. You have been selected to participate in this study because you are involved with cervical cancer patients. This interview will take not more than 30 minutes and you are free to ask me any questions after the interview or if you need any clarity you may stop me during the interview so that I may assist you.

**For HW interviews seek permission from management:** I would like your permission to identify a respondent and begin the interview.

[**Note**: Ensure formal consent process is done prior to interviewing the selected respondent].

| ID01  ID02  ID03  ID04  ID05  ID06  ID07 | IDENTIFICATION Questionnaire No. [ ]  Province   : ______________________________________  District : ______________________________________  Ward : ________________________________________  Type of health facility : **Rural ------------------------1**  **Urban------------------------2**  Health facility Name: __________________________________________  For health facility **Private -----------------------------1**  **Public-------------------------------2** |
| --- | --- |
| ID08 | INTERVIEWER VISITS A B C  Visit 1 Visit 2 Visit 3  DATE ____________ ____________ ______________ |

| **SECTION 1. GENERAL INFORMATION** | | | | | | | | | | Skip instructions | |
| --- | --- | --- | --- | --- | --- | --- | --- | --- | --- | --- | --- |
| **HEALTH WORKER INFORMATION** | | | | | | | | | | | |
| Q101 | | How old were you at your last birthday? | _____________ | | Age in completed years | | | | |  | |
| Q102a | | **Indicate** The Gender Of The Participant. | Female  Male | | 1  2 | | | | |  | |
| Q102b | | What race are you? | Black African  Coloured  White  Asian  Other (specify) ________ | | 1  2  3  4 | | | | |  | |
| Q103 | | What is your profession? | General practitioner  General nurse  Oncology nurse  Nurse aid  Gynaecologist  Radiologist  Oncologist  Pathologist  Physician  Surgeon  Pharmacist  Laboratory Scientist  Medical Physicist  Radiographer  Clinical psychologist  Social worker  Other (specify)_________________ | | 1  2  3  4  5  6  7  8  9  10  11  12  13  14  15  16 | | | | |  | |
| Q104 | | How many years have you been practicing in your profession? | [ ] | | Years | | | | |  | |
| Q104a | | Where did you receive your basic professional training? | University of Zimbabwe  NUST  Midlands State University  Africa University  MoHCC  Foreign institutions  Other (specify)_________ | | 1  2  3  4  5  6 | | | | | For nurses put MoHCC. | |
| Q104b | | Where did you receive your specialist training?  **Multiple responses possible** | University of Zimbabwe  NUST  Midlands State University  Africa University  MoHCC  Foreign institutions  Other (specify)_________ | | 1  2  3  4  5  6 | | | | |  | |
| Q105a | | Have you ever received any on-the job training on cervical cancer treatment and palliative care? | Yes  No | | 1  2 | | | | |  | |
| Q105b | | When was the latest training conducted? | Month Year  [ ] [ ] | |  | | | | |  | |
| Q105c | | Which institution conducted the training? | [ ] | |  | | | | |  | |
| Q105d | | How long was the training? | [ ] | | Days | | | | |  | |
| Q106a | | Do you feel that you have received adequate training to provide cervical cancer treatment and palliative care services? | Yes  No | | 1  2 | | | | |  | |
| Q106b | | If No, provide reasons | [ ] | |  | | | | |  | |
| Q106c | | Does your employer support your continuous professional development (CPD) in cervical cancer treatment and palliative care? | Yes  No  Not applicable | | 1  2  3 | | | | |  | |
| Q107a | | Does your facility have clinical guidelines for the treatment and palliation of cervical cancer patients? | Yes  No  Don’t know | | 1  2  3 | | | | |  | |
| Q107b | | Have you read or heard about **The National Cancer Prevention and Control Strategy for Zimbabwe (2013-2017)**? | Yes  No | | 1  2 | | | | |  | |
| Q107c | | Have you read or heard about the **Zimbabwe Cervical Cancer Prevention and Control Strategy for Zimbabwe (2016-2020)**? | Yes  No | | 1  2 | | | | |  | |
| Q107d | | Do you think Zimbabwe has adequate policies and strategies for the treatment and management of cervical cancer? | Yes  No | | 1  2 | | | | |  | |
| Q107e | | Do you think that the cervical cancer surveillance system is adequate in the Zimbabwe to account for every case? | Yes  No  Don’t know | | 1  2  3 | | | | |  | |
| Q108a | | How many hours do you work in a week? | [ ] | | Hours | | | | |  | |
| Q108b | | How many patients do you attend to daily on average? | [ ] | |  | | | | |  | |
| Q108c | | On average what is your relationship with your patients? | Excellent  Good  Poor  Don’t want to comment | | 1  2  3  4 | | | | |  | |
| Q108d | | Do most of your cervical cancer patients adhere to prescribed treatments? | Yes  No  Don’t know | | 1  2  3 | | | | |  | |
| Q108e | | Do some of your cervical cancer patients miss treatments or other prescribed procedures? | Yes  No  Don’t know | | 1  2  3 | | | | |  | |
| Q108f | | What is the general perception of cervical cancer patients and their families on the services you provide in this facility? | Excellent  Good  Poor  Don’t know | | 1  2  3  4 | | | | |  | |
| Q109a | | How are the working conditions in this facility? | Excellent  Good  Poor  Don’t want to comment | | 1  2  3  4 | | | | |  | |
| Q109b | | Are you motivated to provide your services to cervical cancer patients in this facility? | Yes  No  Don’t want to comment | | 1  2  3 | | | | |  | |
| Q109c | | Do you think the benefits (salaries and allowances) you are receiving are commensurate with the services you provide to cervical cancer patients? | Yes  No  Don’t want to comment | | 1  2  3 | | | | |  | |
| **HEALTH FACILITY INFORMATION** | | | | | | | | | | | |
| Q110 | | Who owns this facility? | Government  NGO  Church based organization  Community  Private for profit  Other (specify) ___________ | | 1  2  3  4  5 | | | | |  | |
| Q111 | | How many health professionals are employed (full or part-time) in this facility? | [ ] | |  | | | | |  | |
| Q112 | | Who mainly pays the salaries of staff at this facility? | Government  NGOs  Church  Own income  Private companies/individuals  Don’t know  Other (specify)_____________ | | 1  2  3  4  5  6 | | | | |  | |
| Q113 | | Who mainly pays for medical supplies for facility? | Government  NGOs  Church  Own income  Private companies/individuals  Don’t know  Other (specify)_____________ | | 1  2  3  4  5  6 | | | | |  | |
| Q114 | | Who mainly pays for running costs for this facility? | Government  NGOs  Church  Own income  Private companies/individuals  Don’t know  Other (specify)_____________ | | 1  2  3  4  5  6 | | | | |  | |
| Q115a | | What is the type of the health facility? | Public hospital  Private Hospital/clinic  Nursing home  Palliative care facility  Other (specify) _______________ | | 1  2  3  4 | | | | |  | |
| Q115b | | Where are do most cervical cancer patients attending this facility come from? [Province] | Manicaland  Bulawayo  Harare  Mashonaland East  Mashonaland Central  Mashonaland West  Midlands  Matebeleland North  Matebeleland South  Masvingo | | 1  2  3  4  5  6  7  8  9  10 | | | | |  | |
| Q116a | | How many professionals serve cervical cancer at your health facility? | General practitioner  General nurse  Oncology nurse  Nurse aid  Gynaecologist  Radiologist  Oncologist  Pathologist  Physician  Surgeon  Pharmacist  Laboratory Scientist  Medical Physicist  Radiographer  Clinical psychologist  Social worker | | [ ]  [ ]  [ ]  [ ]  [ ]  [ ]  [ ]  [ ]  [ ]  [ ]  [ ]  [ ]  [ ]  [ ]  [ ]  [ ] | | | | | Put “00” if the professional is not there. | |
| Q116b | | Are the health professionals adequate to serve the number of patients you receive? | Yes  No | | 1 2 | | | | |  | |
| Q116c | | How many staff members in your speciality should this health facility have to effectively manage your workload? | [ ] | |  | | | | |  | |
| Q116d | | What specialists are in short supplies in this facility?  **Multiple selection possible** | Oncology nurse  Gynaecologist  Radiologist  Oncologist  Pathologist  Physician  Surgeon  Pharmacist  Laboratory Scientist  Medical Physicist  Radiographer  Clinical psychologist  Social worker  Other (specify)_______ | | 1  2  3  4  5  6  7  8  9  10  11  12  13 | | | | |  | |
| Q117a | | How many days per week is this facility open?  **Number 1-7** | [ ] | | Days | | | | |  | |
| Q117b | | How many hours per day (on average) does this facility offer patient consultation/services? | [ ] | | Hours | | | | | If not applicable put 9999 | |
| Q118 | | How many patient visits have you had at this facility in the past 3 months? | [ ] | |  | | | | |  | |
| Q119 | | Does this facility hospitalize cervical cancer patients? | Yes  No | | 1  2 | | | | |  | |
| Q120 | | How many cervical cancer inpatients have you had in the last 3 months in this facility? | [ ] | |  | | | | |  | |
| Q121 | | How many inpatient bed days have you had during the past 3 months? | [ ] | |  | | | | |  | |
| Q122 | | How many beds are available for hospitalization of cervical cancer patients? | [ ] | |  | | | | |  | |
| Q123 | | How many beds in TOTAL are available for patients at your facility? | [ ] | |  | | | | |  | |
| Q124a | | Does this facility offer cervical cancer screening? | Yes  No | | 1  2 | | | | | If No skip to Q125a | |
| Q124b | | Does this facility offer treatment of pre-cervical cancer lesions? | Yes  No | | 1  2 | | | | |  | |
| Q124b | | What treatment options are available in this facility? | Cryotherapy  LEEP  Surgery  Other (specify)___________ | | 1  2  3 | | | | |  | |
| Q125a | | Does this facility offer histology investigations for patients with suspected cervical cancer? | Yes  No | | 1  2 | | | | | If Yes skip to Q126 | |
| Q125b | | If No, where do you refer patients for histological investigations? | Parirenyatwa Hospital  Harare Hospital  Private laboratories  Other (specify) ________ | | 1  2  3 | | | | |  | |
| Q126 | | How long does it take on average for a patient to receive histology results for cervical cancer? | [ ] | | Days | | | | |  | |
| Q127 | | What cervical cancer treatment services are available in your facility?  **Multiple selection possible** | Radiotherapy  Chemotherapy  Radio-chemotherapy  Surgical treatment  Not applicable  Other (specify)___________ | | 1  2  3  4  5 | | | | |  | |
| Q128 | | How long does it take on average for a patient to be started on treatment? | [ ] | | Days | | | | |  | |
| Q129 | | How many cervical cancer patients are registered in this facility? | [ ] | |  | | | | |  | |
| Q130a | | Of these patients how many are on treatment? | [ ] | |  | | | | |  | |
| Q130b | | How many cervical cancer patients were put on treatment in the last 3 months? | [ ] | |  | | | | |  | |
| Q131a | | How many patients are on palliative care in this facility? | [ ] | |  | | | | |  | |
| Q131b | | How many patients were put on palliative care in the last 3 months? | [ ] | |  | | | | |  | |
| Q132 | | How many patients were put on treatment in the last 3 months? : | Radiotherapy  Chemotherapy  Radio-chemotherapy  Surgical treatment  Other (specify)___________ | | [ ]  [ ]  [ ]  [ ] | | | | |  | |
| Q133 | | Does this facility transfer/refer patients to other facilities? | Yes  No | | 1  2 | | | | | If No skip to Q136a | |
| Q134 | | Where do you usually transfer/refer patients for other services?  **Multiple selection possible** | Parirenyatwa Hospital  Harare Hospital  Private hospitals/clinics  Private laboratories  Other(specify)________________ | | 1  2  3  4 | | | | |  | |
| Q135 | | What services do you usually transfer/refer patients for?  **Multiple selection possible** | Laboratory services  Treatment  Palliative care  Radiology  Other (specify)____________ | | 1  2  3  4 | | | | |  | |
| Q136a | | Do you think most patients diagnosed of cervical cancer have access to treatment and palliative care? | Yes  No | | 1  2 | | | | |  | |
| Q136b | | How are most patients paying for cervical cancer treatment or palliative care in this facility? | Own funds  Medical aid  NGOs  Private donors  Other(specify)___________ | | 1  2  3  4 | | | | |  | |
| Q136c | | Are medical aid schemes fully covering cervical cancer treatment and palliative care in Zimbabwe? | Yes  No  Don’t know | | 1  2  3 | | | | |  | |
| Q137 | | What challenges do you think most patients experience in accessing and utilizing treatment and palliative care services?  **Multiple selection possible**  **Probe for more challenges** | Lack of transport to go the health centres.  Lack of finances to pay for the services.  Few centres offer the specialized services.  Bad attitude of health professionals  Stigma from the society  Stock-outs of drugs (medication) at health facilities.  Bureaucratic processes in accessing treatment services  Lack or dysfunctional equipment at health centres.  Other (specify)____________ | | 1  2  3  4  5  6  7  8 | | | | |  | |
| Q138 | | How do you think these challenges could be overcome to improve access and utilization of cervical cancer treatment and palliative care services?  **Multiple responses possible** | Provide free services  Government to build more health facilities.  Government to help patients get treatment abroad.  Government to train and hire more health workers.  NGOs to establish treating and palliative care centres.  Government to increase capacity in existing health facilities.  More funding to health facilities.  Other (specify_________ | | 1  2  3  4  5  6  7 | | | | |  | |
| **INFRASTRUCTURE** | | | | | | | | | | | |
| **Electricity and Power** | | | | | | | | | | | |
| Q201 | What is the main source of electricity for the facility | | Mains  Generator  Solar panel  No power supply  Other (specify)___________ | | | 1  2  3  4 | | | |  | |
| Q202 | Over the past 3 months have you experienced any power interruptions of this source of more than 2 hours? | | Yes  No | | | 1  2 | | | | If no skip to Q205 | |
| Q203 | How long was the longest interruption? | | [ ] hours | | |  | | | |  | |
| Q204 | In the last 2 weeks how many days was electricity from this source interrupted for more than 2 hours at a time? | | [ ] days | | |  | | | |  | |
| Q205 | Does this facility have a back-up source of electricity?  **Multiple selection possible** | | Mains  Generator  Solar panels  Inverter  None  Other (specify) __________ | | | 1  2  3  4  5 | | | |  | |
| **Water and sanitation** | | | | | | | | | | | |
| Q206 | What is the main source of water for this facility? | | Piped from mains  Borehole  Tanker  Bottled water  Other (specify)____________ | | | | 1  2  3  4 | |  | | |
| Q207 | Over the last 3 months have you experienced water supply interruptions of this source of more than 2 hour? | | Yes  No | | | | 1  2 | | If no skip to Q210 | | |
| Q208 | How long was the longest interruption? | | [ ] hours | | | |  | |  | | |
| Q209 | Over the past 2 weeks, how many days was water from this source interrupted for more than 2 hours at a time? | | [ ] Days | | | |  | |  | | |
| Q210 | Does this facility have a back-up water supply  **Multiple selection possible** | | Piped from mains  Borehole  Tanker  Bottled water  Other (specify)____________ | | | | 1  2  3  4 | |  | | |
| Q209 | What type of toilet is available for patients? | | **Flush or pour flush toilet**  Flush to piped sewer system  Flush with septic tank  Flush to pit latrine  Flush to somewhere else  Flush, don’t know where  **Pit latrine**  Ventilated improved pit latrine  Pit latrine with slab  Pit latrine without slap/open pit  Bucket toilet  No facility/bush/field  Other (specify)___________ | | | | 1  2  3  4  5  6  7  8  9  10  11 | |  | | |
| Q210 | How many toilets are available in the facility? | | **[ ]** | | | |  | |  | | |
| Q211 | How many of the mentioned toilets are currently functioning? | | **[ ]** | | | |  | |  | | |
| Q212 | What is the overall hygiene condition of patient toilets?  **Observe if possible** | | Extremely clean and well maintained  Reasonable clean and maintained  Not very clean or maintained | | | | 1  2  3 | |  | | |
| Q213 | Are there separate toilets for male and female patients? | | Yes  No | | | | 1  2 | |  | | |
| Q214 | Are there functional (soap and water) hand washing facilities for patients or in the toilets? | | Yes  No | | | | 1  2 | |  | | |
| Q215 | What method does this facility use in the final disposal of sharps?  **Multiple selection possible** | | **Open burning with the facility**  Flat ground-no protection  Pit or protected ground  **Dump without burning (within facility)**  Flat ground-no protection  Covered pit or pit latrine  Open pit-no protection  Protected ground or pit  **Remove off site**  Stored in covered container  Stored in other protected environment  Stored unprotected  Burn incinerator  Other (specify)____________ | | | | 1  2    3  4  5  6  7  8  9  10 | |  | | |
| Q216 | Is the incinerator functional today? | | Yes  No | | | | 1  2 | |  | | |
| Q217 | Is the power source for the incinerator available today? | | Yes  No | | | | 1  2 | |  | | |
| Q218 | Does the facility have guidelines for health care waste management? | | Yes  No | | | | 1  2 | |  | | |
| Q219 | Have you or any staff member received training in health care waste management practices in the past 2 years? | | Yes  No | | | | 1  2 | |  | | |
| **Communication** | | | | | | | | | | | |
| Q220a | | Does the health facility have:  **Observe if possible** | | Fixed telephone  Mobile phone for facility  Mobile phone for staff useable by facility  Computer for facility  Short wave radio  Internet access  Health information systems  . | | | | No Yes  0 1  0 1  0 1  0 1  0 1  0 1  0 1 | | |  |
| Q220b | | Does your facility have a system to document and monitor the treatment of cervical patients on a regular basis? | | Yes  No  Don’t know | | | | 1  2  3 | | |  |
| **Emergency Transportation and Ambulance services** | | | | | | | | | | | |
| Q221 | | Does the facility have access to ambulance facility for emergency transport? | | Yes  No | | | | 1  2 | | |  |
| Q222 | | If the facility owns an ambulance is fuel available for use in cases of emergency? | | Yes  No  Not applicable | | | | 1  2  3 | | |  |
| Q223 | | Who pays for ambulance services in emergency situations? | | Health facility  Patients/ Medical aid  Other (specify) ______________ | | | | 1  2 | | |  |
| Q224 | | Has the facility faced challenges in transporting patients in emergency situations in the last 3 months? | | Yes  No | | | | 1  2 | | |  |
| **EQUIPMENT, MATERIALS AND SUPPLIES** | | | | | | | | | | | |
| Q225 | | Does the health facility have adequate basic equipment? | | Yes  No | | | | 1  2 | | |  |
| Q226 | | Is most equipment in this facility in functional order? | | Yes  No | | | | 1  2 | | |  |
| Q227a | | In the last 3 months how many major equipment breakdowns has the facility experienced? | | [ ] | | | |  | | |  |
| Q227b | | How long did it take for the equipment to repaired or replaced? | | [ ] | | | | Days | | |  |
| Q228 | | Does the facility have back up equipment for use in cases of major equipment breakdowns? | | Yes  No | | | | 1  2 | | |  |
| Q228 | | Does the facility have modern equipment for treating cervical cancer? | | Yes  No | | | | 1  2 | | |  |
| **DRUGS AND CONSUMABLES** | | | | | | | | | | | |
| Q229 | | Does the facility have adequate stocks of drugs such as cisplastin for treatment of cervical cancer today? | | Yes  No | | | | 1  2 | | |  |
| Q230 | | Did the facility experience stock-outs of cisplastin in the last 3 months? | | Yes  No | | | | 1  2 | | |  |
| Q231 | | How long did the stock-out last? | | [ ] | | | | Days | | |  |
| Q232 | | Does the facility have adequate analgesics and other medication for palliative care patients today? | | Yes  No | | | | 1  2 | | |  |
| Q233 | | Did the facility experience stock of outs of analgesics and other medication for palliative care patients in the last 3 months? | | Yes  No | | | | 1  2 | | |  |
| Q234 | | What contingency plans does the health facility have for major drugs supplies for cervical cancer treatment and management of palliative care patients? | | None  Patient have to buy  Borrow from other facilities  Never experience stock-outs  Other (specify)_____________ | | | | 1  2  3  4  5 | | |  |

**Interviewer’s observations**

**___________________________________________________________________________________________________________________________________________________________________________________________________________________________________________________________**

**Comments on specific questions ________________________________________________________________________________________________________________________________________________________________________Any other comments ___________________________________________________________________________________________________________________________________________________________________________________________________________________________________________________________**

**Remarks:** Thank the participant for their time and proceed to the next respondent.

**----------------------------------------------------------------THE END---------------------------------------------------------------**
