# Supplementary material for: Health system constraints affecting treatment and care among women with cervical cancer in Harare, Zimbabwe
Source: BMC Health Serv Res. 2019 Nov 12;19:829. doi: 10.1186/s12913-019-4697-6 (PMC6852958; doi:10.1186/s12913-019-4697-6)
Supplement: Supplementary file 3 — Additional file 3. In-depth interview guide [English and Shona]. [file 12913_2019_4697_MOESM3_ESM.docx]

**IN-DEPTH INTERVIEW GUIDE**

| **DISCUSSION QUESTIONS** |
| --- |
| **Note that the subsequent questions are a guide to the qualitative interview: they do not all have to be asked and the interviewer may modify the order and ask additional probing questions. Questions with “[C]’’ are applicable to cervical cancer survivors while those marked “[H]” will be applicable to healthy women and men.** |
| **General information**   - Can you tell me about yourself? Probe for :whether they are cervical cancer survivor, healthy woman or a man, race, place of residence, age, marital status and religion   *Ndinokumbira kuti mundiudze nezvenyu. Bvunza kuti munhu akararama shure kwekuva ne gomarara remuromo wechibereko, munhukadzi, munhurume, rudzi, nzvimbo yaanogara, makore ekuberekwa, mamiriro ake ekuroorwa/kuroora, ne chitendero chake?* |
| **Knowledge of cervical cancer**   - Tell me what you have heard about cervical cancer? [H]   *Mukandi udze zvamakambonzwa nezve gomarara remuromo we chibereko*   - Before you were diagnosed of your condition, can you please tell me what you heard about it? [C]   *Musati marwara manga mambonzwa nezve gomarara iri?*   - Can you please tell me where you have heard about cervical cancer and what you heard about it?   *Mungandiudze kuti makatanga kunzwa kupi nezve gomarara remuromo wechibereko?*   - Can you please tell me more about what how you were diagnosed and where? Probe for place and dates (month, year) [C]   *Mungandiudze zvizere pamusoro pekuti makabatwa sei, uye muri kupi kuti mune gomarara remuromo wechibereko? Bvunza pamusoro penzvimbo ne mazuva ( mwedzi ne gore)*   - To your knowledge what are some of the causes of cervical cancer?   *Mukuziva kwenyu ndezvipi zvinokenzera gomarara remuromo wechibereko?*   - Tell me how you would prevent cervical cancer? Probe for screening and treatment of precancerous lesions, vaccination and male circumcision of partners.   *Mungandiudze kuti gomarara remuromo wechibereko ringadzivirirwe sei? Bvunza pamusoro pekuongororwa nekurapwa kwezvironda zvinovapo gomarara risati ratanga, kubayiwa mishonga yekudzivirira ne kuchecheudzwa kwevarume?*   - Tell me some of the signs and symptoms of cervical cancer?   *Mungandiudze kuti ndezvipi zvinoratidza nezvino nzwikwa nemhunu anegomarara remuromo wechibereko?*   - Can you tell me what you think about cervical cancer risk in your community and your reasoning behind your thoughts?   *Mungandiudze zvamunofunga pamusoro pekuti vanhu vemunharaunda yenyu varipanjodzi yekuva ne gomarara remuromo wechibereko? Uye sei muine mafungiro iwayo?* |
| **Experiences of cervical cancer**   - Can you tell me about your experiences with cervical cancer in your household/community? [H]. Probe for dates, age and relationship to participant.   *Mungandiudze kuti zvii zvamakasangana nazvo mumhuri yenyu /nharaunda yenyu zvakanganan negomarara remuromo wechibereko? Bvunza pamusoro pemazuva, makore ekuberekwa ne hukama*   - Can you tell me more about your diagnosis history for your conditions? [C]   *Mungandiudze zvizere pamusoro pekurarwa nekurapwa kwenyu?*   - Probe for name of health facility, dates and health professionals involved?   *Bvunza zita rekirinika/chipatara, mazuva ne vekumakiriniki/zvipatara vari kurapa murwere?*   - What caused you to go the health facility where you were diagnosed? Probe for reasons such as routine screening, health problems, health worker advice, friend or relative advice? [C]   *Ndezvipi zvikonzero zvakaita kuti muende kukiriniki/ chipatara kwamakano udzwa kuti mune gomarar remuromo wechibereko? Bvunza pamusoro pezvikonzero zvakaita sekuenda kuno ongororwa nguva ne nguva, matambudziko ehutano, kukurudzirwa nevanoshanda kuzvupatara, ne shamwari kana hama?*   - Tell me what you know about treatment and palliative care services for cervical cancer? Probe for sources of information?   *Mungandiudze zvamunoziva nezve kurapwa nerutsigirwo runopiwa nevanochengeta vanegomarara remuromo wechibereko? Bvunza kuti ruzivo uru akaruwana kupi?*   - Can you tell me what you know about support from partners, friends and families for cervical cancer patents in your community?   *Mungandiudze zvamunoziva pamusoro pe rutsigiro runopiwa vanhu vane gomarara remuromo wechibereko, kubva kune varume vavo, shamwari dzavo pamwe ne mhuri dzavo munharaunda yenyu?*   - How do you think partner, friend or family support may help cervical cancer patients?   *Munofunga kuti vanhu vanegomarara remuromo wechibereko vanowana rutsigiro rwupi kubva kuvarume vavo, shamwari dzavo pamwe ne mhuri dzavo*   - What roles are churches in Harare playing to support cervical cancer patients? Probe for social, emotional and spiritual support. What are the general perceptions of people in your community with regards to cervical cancer? Probe for attitudes, beliefs, misconceptions and fears?   *Machechi emu Harare arikuita zvipi mukupa rutsigiro kune vane gomarara remuromo wechibereko? Bvunza pamusoro perubatsiro runechekuita nemagariro, zvavanonzwa ne mweya. Bvunza pamusoro pe mafungiro akajairika munharaunda yenyu akanangana ne gomarara remuromo wechibereko. Bvunza pamusoro pemafungiro, zvavanotenda, zvavazinga zwisise ne zvavanotya.* |
| **Access to cervical cancer treatment and palliative care**   - Which health facilities that you know or have heard about provide cervical cancer treatment and palliative care in Harare?   *Ndekupi muHarare kwamunoziva kana kwamakanzwa kuti kuno rapwa gomarara remuromo wechibereko kana kuno wanikwa rubatsiro kune vanochengeta vane gomarara?*   - Tell me about access to cervical cancer diagnosis, treatment and palliative care services in your community?   *Munofunga kuti vanhu vemunharaunda yenyu vanokwanisa kuwana rubatsiro nekuongororwa uye nerutsigiro kune vanochengeta vano rwara negomarara?*   - Tell me more about what you know or have heard about how cervical cancer is treated in health facilities?   *Mungandiudze zvizere pamusoro pe zvamunoziva kana zvamakambonzwa maererano nekuti gomarara remuromo wechibereko rino rapwa sei mumakirniki/zvipatara?*   - Who do you think can best treat and manage cervical cancer and why you think so? Probe for health workers, traditional healers, pastors and prophets?   *Ndiyani wamunofunga kuti anogona kurapa gomarara remuromo wechibereko zviri nani? Bvunza pamusoro pe kuti kirinika/chipatara , n’anga kana kuti vaporofita?*   - Tell me about what you think about the fees charged for cervical cancer treatment and palliative services in your community? Probe for affordability.   *Mungandi udze zvamunofunga pamusoro pe mari dzinodiwa kuti munhu arapwe kana kuti awane rubatsiro rune chekuita nekuchengetwa kwe vane gomarara remuromo wechibereko munharaunda yenyu? Bvunza pamusoro pekukwanisa kwevanhu kubhadara.*   - What do you think about the availability of treatment services in Harare are adequate to cover all cervical cancer patients? Probe for reasons of response?   *Munofunga kuti zvipatara /makiriniki zvi/anowanikwa muHarare zvinorapa vanhu vanoda rubatsiro rwakadai zvakakwana? Bvunza zvekonzero zvaita kuti ape mhinduro yakadai.*   - What do you think about the adequacy of doctors and specialists to treat cervical cancer in Harare? Probe for reasons of response?   *Munofunga kuti mu Harare anachiremba na anamazvikokota vanorapa gomarara remuromo wechiberoko vanokwana? Bvunza pamusoro pemhinduro yawapiwa?*   - What are your opinions on the training of doctors and specialists to provide good treatment services to cervical cancer patients?   *Munofunga kuti anachiremba na anamazvikokota vakadzidziswa zvakakwana kuti varape vanhu zvakanaka?*   - What do you know about palliative care in health facilities in Harare? Probe for names of some of the facilities?   *Ndekupi muHarare kwamunoziva kuno wanikwa rubatsiro kune vanochengeta vane gomarara? Bvunnza mazita enzvimbo idzodzo?*   - Tell me what you know/think about the adequacy of palliative services to cover the patients who need such services?   *Munofunga kuti zvipatara /makiriniki zvi/anorapa vanhu vanoda rubatsiro rwakadai zvakakwana?*   - Describe some of the challenges that you/partner or other member of your family or community have experienced or experience in seeking cervical cancer treatment or palliative care?   *Mungatsanangure kuti ndeapi matambudziko amunosangana nawo /kana anosangana nemumwe wenyu kana kuti vanhu vemunharaunda yenyu kana vachida kuno rapwa kana kuti kunowana rubatsiro kune avo vano chengeta vanegomarara mu munharaudna yenyu?* |
| **Utilization of cervical cancer treatment and palliative care**   - If you or your partner or relative were diagnosed of cervical cancer what would you do? [H] Probe for reasons of response and where you would go for treatment?   *Imi kana kuti mumwe wenyu kana hama yenyu ikabatwa gomarara remuromo wechibereko mungaite sei? Bvunza pamusoro pezvikonzero zvaita kuti ape mhinduro iyoyo uye kuti mungaende kupi kunorapwa?*   - Tell me more about your experiences with cervical cancer treatment from the time of diagnosis (histologically confirmed)? [C] Probe for health facility, traditional healer or prophet and frequency of health facility visits?   *Mungandiudze zvizere pamusoro pe zavamakasangana nazvo mukurapwa gomarara remuromo wechibereko, kubva paguva yeku ongororwa (mumalebhu)? Bvunza pamusoro pekiriniki/chipatara, na’nga, kana muporofita nekuti vaenda kukiriniki/chipatara kangani?*   - What do you think about the experiences of women with cervical cancer in getting treatment in Harare? Probe for reasons of response?   *Munofungei pamusoro pezvinosangana nevakadzi vane gomarara remuromo wechibereko kana vachirapwa muHarare? Bvunza zvikonzero zvaita kuti ape mhinduro iyoyo?*   - Can you tell me if there are instances you have you missed appointments or medication for your treatment and if so what were your reasons? [C]   *Mungandiudze kana Mmakamboregedza kuenda kwachiremba kana kutora mishonga yenyu uye zvikonzero zvakaita kutizvidaro.*   - What are your opinions of the effectiveness of the treatment you are receiving? Probe for reasons response? [C]   *Mawonero enyu takatarisa kurapwa kwamuri kuitwa, mungati kuri kukushandirai? Bvunza pamusoro pezvikonzero zvaita ita kuti ape mhinduro iyoyo.*   - Tell me your opinions about the effectiveness of treatments given to cervical cancer patients at health facilities? Probe for reasons? [H]   *Munofunga kuti kurapwa kurukitwa avo vane gomarara remuromo wechibereko kunoshanda here? Bvunza pamusoro pezvikonzero?*   - What challenges have you/your partner faced in using treatment or palliative services for your condition? [C]   *Ndeapi matambudziko amakasanga nawo kana akasangana nemumwe wenyu mukushandisa mushonga kana kuwana rubatsir o runopiwa vane gomarara remuromo wechibereko*   - What do you think are some of the challenges that cervical cancer patients and their families are facing in using treatment and palliative care services in Harare? [H]   *Munofunga kuti ndeapi matambudziko anosangana ne avo vanegomarara remuromo wechibereko kana vachida kuno rapwa kana kuti kunowana rubatsiro kune vano chengeta vanegomarara mu Harare?*  **Health services**   - Can you tell me more about your nearest health facility? Probe distance, time taken to get to the facility and mode of transport to the facility?   *Mungandiudzei zvizere pamusoro pekiriniki kana chipatara chamungati chiripaduze nemusha wenyu? Bvunza pamusoro pekuti kure zvakadii ne nguva inotorwa kuti vasvike kukiriniki/chipatara*   - What information have you received from your health facility with regards to cervical cancer?   *Ndeapi mashoko akanangana nezve gomarara remuromo wechibereko amakawana kubva kukiriniki /kana chipatara*   - If you or a member of your household were to have signs and symptoms of cervical cancer, what would you do and why?   *Kana munhu wemunharaunda ino akava nezvino ratidza kana kunzwa zvino nzwikwa nevane gomarar remuromo we chibereko, angaite sei? Sei madaro?*   - What are your opinions of people in your community with you regards to seeking treatment early when they suspect cervical cancer?   *Munofunga kuti vanhu vemunharaunda yenyu vanotsvaga rubatsiro nguva ichiripo kana vachifungidzira kuti vane gomarara remuromo wechibereko?*   - What are your opinions about adequacy of equipment, drugs, beds and other infrastructure to provide cervical treatment and palliative care in health facilities in Harare? [C]   *Mukufunga kwenyu makiriniki/zvipatara zvine zvakakwana zvakaita se mishini, mibhedha, mishonga ne zvimwe zvakadaro zvinoshandiswa mukurapa nekuchengeta vane gomarara remuromo wechibereko*   - What are your opinions about the adequacy of health workers (nurses, nurse aids, doctors, specialists, pharmacists, radiographers and laboratory scientists) to provide treatment and palliative care of cervical cancer patients in health facilities in Harare? [C]   *Munofunga kuti makiriniki/zvipatara zvine vashandi vakakwana (anamukoti, anachiremba, anamazvikokota, vanopa mishonga nevano shanda mumalebhu) vakakwana uye vachikwanisa kurapa nekuchengeta vane gomarara remuromo wechibereko?*   - Can you tell me more about your opinions on the fees that are charged for treatment services by your health facility? [C]   *Mungandiudze zvizere pamusoro pemafungiro enyu pamari dzinodiwa kuti vanhu varapwe mumakiriniki kana zvipatara?*   - To your knowledge what are the experiences of patients who cannot pay or do not afford to pay for treatment or other procedures at health facilities?   *Mukuziva kwenyu varhwere vasingakwanise kubhadara mari dzinodiwa kukiriniki /chipatara kuti varapwe vanosangana nei?*   - What are your perceptions on the quality of services provided at this/your health facility? Probe for attitudes of nurse aids, nurses, doctors, specialists, radiographers, pharmacists and other health workers?   *Mukufunga kwenyu mungati mhando yekurapwa/mashandirwo emuma kiriniki/zvipatara yakamirasei? Bvunza pamusoro pemafungiro evakoti, anachiremba, anamazvikokota, nevanopa mishonga nevamwe vashandi vemuzvipatara?*   - In your opinion what are the perceptions and attitudes of your local leadership (herdmen, counsellors, and chiefs) have on people using health services when they are sick or for routine checkups?   *Mukuwona kwenyu, akamira sei mafungiro evatungamiri venharaunda yako ( mahedhimeni, makhansela na namambo) zvakangana nekuenda kwevanhu kuzvipatara/makiriniki kana vachirwara kana kuti kuno ongororwa zvisinei kuti havazi kurwara?.*   - What do you think should be done at this facility and generally in Zimbabwe to improve cervical cancer treatment and palliative care services?   *Ndezvipi zvamunofunga kuti zvinofanirwa kuitwa muzvipatara kana makiriniki kuvandudza kurapwa kwegomarara remuromo wechibereko ne kuchengetwa /kubatsirwa kwe vane gomarara iri mu Zimbabwe?* |

**Remarks:** Thank the participant for their time and proceed to the next participant.

**--------------------------------------------The End------------------------------------------------------**
